# Physiological and Genetic Regulation for High Lipid Accumulation by *Chlorella sorokiniana* Strains from Different Environments of an Arctic Glacier, Desert, and Temperate Lake under Nitrogen Deprivation Conditions

**DOI:** 10.1128/spectrum.00394-22

**Published:** 2022-10-06

**Authors:** Shanmei Zou, Zheng Huang, Xuemin Wu, Xinke Yu

**Affiliations:** a Jiangsu Provincial Key Laboratory of Marine Biology, College of Resources and Environmental Science, Nanjing Agricultural University, Nanjing, P. R. China; Technical University of Denmark

**Keywords:** microalgae, extreme environments, lipid accumulation, physiological process, genetic regulation, nitrogen deprivation

## Abstract

Microalgae can adapt to extreme environments with specialized metabolic mechanisms. Here, we report comparative physiological and genetic regulation analyses of *Chlorella sorokiniana* from different environmental regions of an arctic glacier, desert, and temperate native lake in response to N deprivation, for screening the optimal strain with high lipid accumulation. Strains from the three regions showed different growth and biochemical compositions under N deprivation. The arctic glacier and desert strains produced higher soluble sugar content than strains from the native lake. The arctic glacier strains produced the highest levels of lipid content and neutral lipids under N deprivation compared with strains from desert and native lake. At a molecular level, the arctic strain produced more differentially expressed genes related to fatty acid biosynthesis, glycolysis gluconeogenesis, and glycerolipid metabolism. The important functional genes acetyl coenzyme A (acetyl-CoA) carboxylase (ACCase), fatty acid synthase complex, pyruvate dehydrogenase component, and fatty acyl-acyl carrier protein (acyl-ACP) thioesterase were highly expressed in arctic strains. More acetyl-CoA was produced from glycolysis gluconeogenesis and glycerolipid metabolism, which then produced more fatty acid with the catalytic function of ACCase and acyl-ACP thioesterase in fatty acid biosynthesis. Our results indicated that the *C. sorokiniana* strains from the arctic region had the fullest potential for biodiesel production due to special genetic regulation related to fatty acid synthesis, glycolysis gluconeogenesis, and glycerolipid metabolism.

**IMPORTANCE** It is important to reveal the physiological and genetic regulation mechanisms of microalgae for screening potential strains with high lipid production. Our results showed that *Chlorella sorokiniana* strains from arctic glacier, desert, and temperate native lake had different growth, biochemical composition, and genetic expression under N deprivation. The strains from an arctic glacier produced the highest lipid content (including neutral lipid), which was related to the genetic regulation of fatty acid biosynthesis, glycolysis gluconeogenesis, and glycerolipid metabolism. The functional genes for acetyl-CoA carboxylase, fatty acid synthase complex, pyruvate dehydrogenase component, and fatty acyl-ACP thioesterase in the three pathways were highly expressed in arctic strains. The revelation of physiological and genetic regulation of strains from different environmental regions will contribute to the microalgae selection for high lipid accumulation.

## INTRODUCTION

Overconsumption of fossil fuel by industry and transportation leads to decreasing world fossil fuel reserves and serious environment problems, e.g., atmospheric pollution and global warming (1). It is important to search for alternative and ecologically friendly energy resources. Recently, biodiesel, which can be produced from a variety of sources (including plants, animals, and microbes) has been proposed as a renewable energy source (2). The search for sustainable sources of biofuels has led to renewed interest in microalgae as a potential feedstock, due to the potential to synthesize and accumulate large quantities of lipids in some species (3, 4). The advantage of microalgae is that they can accumulate large quantities of oils in the form of triacylglycerols (TAGs), which are preferred renewable oils because they possess a high molar ratio of hydrogen to carbon (4–9). Microalgae grown under nutrient limitation exhibit considerable variation in their biochemical composition, depending on the limiting nutrient and the degree of limitation. The nitrogen deprivation response is perhaps the best-characterized inducer of lipid accumulation in microalgae (10–13). In addition to increased total lipid content, N deprivation can also induce changes in fatty acid chain length and saturation (14, 15). Moreover, some species of microalgae can be efficiently transformed, which makes it possible to enhance the productivity of natural compounds through genetic strain engineering strategies (16, 17).

Among the oleaginous microalgae, the *Chlorella* genus of green algae (*Chlorophyceae*) is commonly considered a promising candidate, due to its high photosynthetic efficiency, lipid productivity, and fast growth ability (18). *Chlorella* microalgae are able to adapt to a variety of environmental conditions, including extreme habitats ranging from desert soil to the polar region (19). Although *Chlorella* has proved to be a potential industrial microalgae for biological engineering, it is unclear whether strains from some environments are more suitable for producing high levels of lipids (15, 20–24).

At present, screening oleaginous microalgae strains which have great potential to produce TAGs is of great importance for biodiesel production. The potentially oleaginous strains could also be used in transgenic microalgae, which could be used as industrial microalgae based on biological engineering (25). This biological engineering technology shows great promise to simplify the production process and significantly decrease biodiesel production costs. Many microalgae can adapt to extreme habitats ranging from desert soil to arctic regions (19, 26). Their long-term adaptation shows that the microalgae can adjust their physiological responses to adapt to such environments (27). In particular, microalgae from extreme environments possess special physiological features and molecular mechanisms for their adaption (28). In this context, microalgae strains in various environments may have different physiological and genetic regulation metabolisms, including those for growth and lipid accumulation. For example, it was indicated that the increased total lipid content and fatty acid composition of Antarctic microalgae could provide its adaptability to low temperatures (29). Some desert microalgae have also been shown to have lipid productivity (30). Therefore, comparative analyses of microalgae strains from different environmental regions in response to nitrogen stress may be used to screen the potential industrial microalgae for biofuel production and also be used in transgenic microalgae for biofuel production. Presently, few studies have compared microalgae from extreme and native environmental regions, e.g., polar, desert, and temperate regions.

Here, we report the growth, lipid accumulation, and transcriptome analysis of three Chlorella sorokiniana strains from different environmental regions, an arctic glacier, desert soil, and temperate native lake, in response to nitrogen stress. The responses in terms of their cell growth, biochemical compositions, lipid productivity, fatty acid profiles, and functional genetic expression to N deprivation were compared. We aimed to reveal the physiological and genetic regulation of microalgae from different environmental regions with N deprivation for selecting potential industrial microalgae and providing the basis for transgenic microalgae in biofuels.

## RESULTS AND DISCUSSION

### Isolation and identification of *Chlorella* strains from different environmental regions of arctic glacier, desert soil, and temperate native lake.

Microalgal biomass, a promising source of biofuel, could contribute to the decrease in our dependence on fossil fuel while offering multiple environmental advantages compared with traditional biofuel land crops. Long-term evolution has made possible the development in algae of physiological adaptations to cope with different natural environments (31). It was found that nitrogen deprivation plays an important role in lipid accumulation of microalgae (11–13). It is possible to screen microalgae strains with high lipid production under N deprivation from different environmental regions by comparing their physiological and genetic regulation mechanisms. *Chlorella*, a kind of oleaginous microalgae, is one of the genera of microalgae that has achieved commercial success on a large scale and can occupy extreme habitats. In the present research, three different *Chlorella* sp. strains were isolated from arctic glacier (referred to here as *Chorella*-Arc), desert soil (*Chorella*-Des), and temperate native lake (*Chorella*-Nat) regions. They were first identified based on morphological characteristics. Then, both *rbcL* and *tufA* sequences were obtained from the three strains and were analyzed using BLAST against sequences in GenBank.

**FIG 1:**
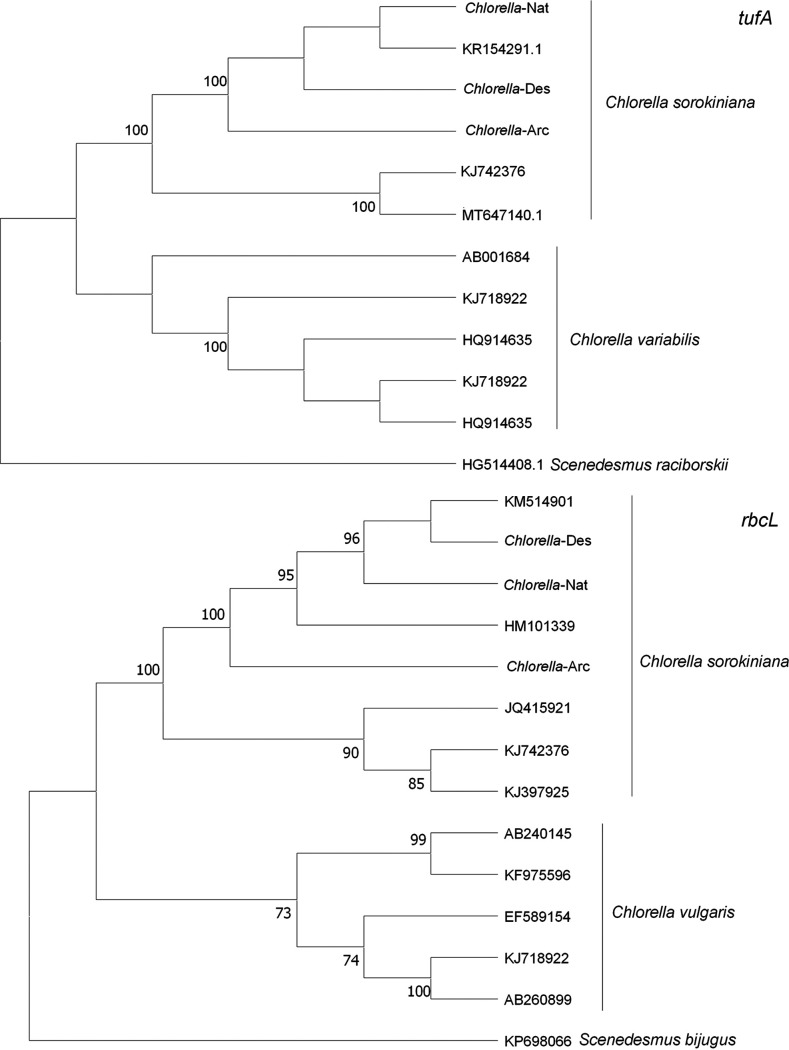
Maximum-likelihood trees of *Chlorella* strains inferred from *tufA* and *rbcL* gene sequences (the three strains used in this study are underlined). Data are based on 1,000 replications; bootstrap values of >50% are shown above the internodes.

The identification of these *Chlorella* sp. strains from the three environmental regions is illustrated in Fig. 1. The corresponding GenBank accession numbers are as follows: for *tufA*, KR154271.1 (*Chlorella*-Nat), KR154255.1 (*Chlorella*-Des), and ON602074 (*Chlorella*-Arc); for *rbcL*, KM514865.1 (*Chlorella*-Nat), KM514884.1 (*Chlorella*-Des), and ON602073 (*Chlorella*-Arc). It was shown that the three strains clustered together with other *Chlorella sorokiniana* strains (from published sequences) in the *tufA* phylogenetic tree, which was independent from the Chlorella variabilis clade. The three strains also clustered together with other published *C. sorokiniana* sequences in the *rbcL* phylogenetic tree. Although *Chlorella*-Arc was not much closer to *Chlorella*-Des and *Chlorella*-Nat, it was in the same clade with the other two *C. sorokiniana* sequences. Thus, the results indicated that the three strains from desert, template native lake, and arctic glacier were identified as *C. sorokiniana* by molecular tools.

### Effect of nitrogen deprivation on growth of the three *C. sorokiniana* strains.

**FIG 2:**
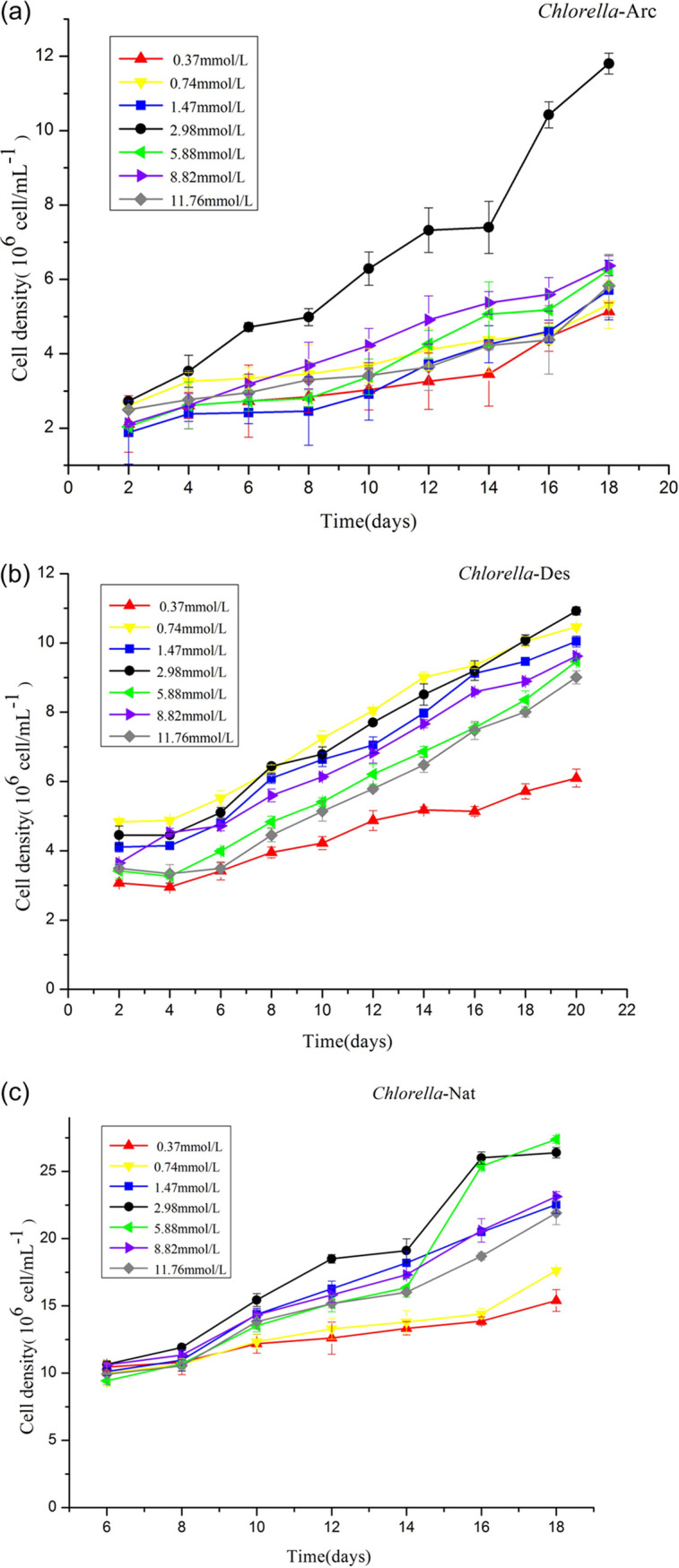
Growth curves of *Chlorella*-Arc (a), *Chlorella*-Des (b), and *Chlorella*-Nat (c) cultured under different sodium nitrate concentrations. The points represent means ± SD of triplicate samples.

The growth rates of the three *C. sorokiniana* strains from arctic glacier, desert, and template native lake in the medium deprived of different nitrogen are shown in Fig. 2, with numbers of cells provided. Generally, a lower or higher nitrogen concentration than 2.98 mmol/liter did not promote growth for the three strains. *Chlorella*-Des grew well in 0.74 mmol/liter, which may have been due to the extremely scarce nutrition in a desert. In addition, the growth of the three strains seemed to be region dependent under both N deprivation and N-replete cultural conditions. By comparison, the strain from the arctic glacier was more nitrogen sensitive than was the temperate native lake algae. The reason for the difference growth rates may be that the strains in different environmental regions have distinct mechanisms of growth adaptations and oil accumulation. So far, the mechanism of oil accumulation in microalgae, including N gene expression of nitrogen starvation-induced accumulation, is still not clear (13, 24, 32). The distinct responses to N deprivation and N-replete conditions among strains from different environmental regions indicated that microalgae could perform special adaptation to the nutritional limits in a regional environment, which is significant when selecting high-quality strains for producing biodiesel, especially based on genetic engineering.

### Effect of nitrogen deprivation on biochemical composition.

**FIG 3:**
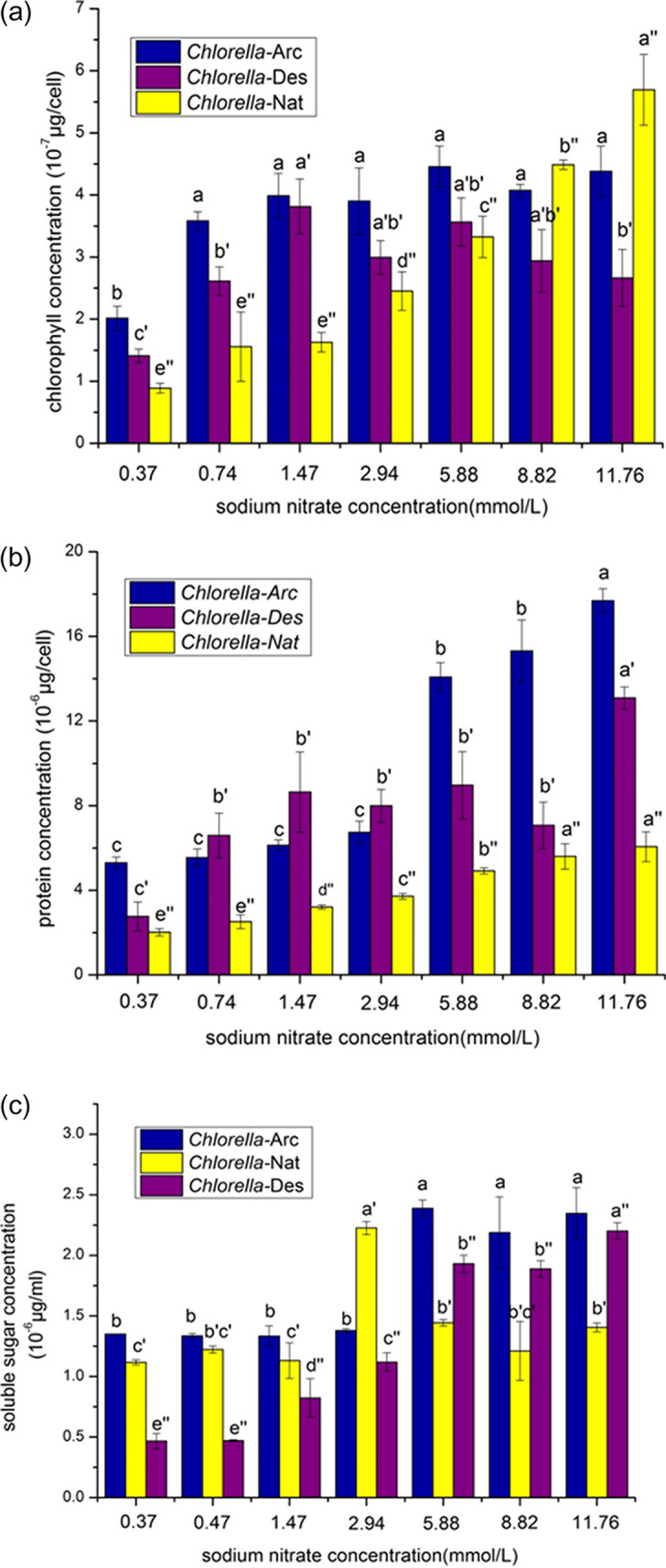
The changes of chlorophyll (a), protein (b), and soluble sugar (c) concentrations of *Chlorella*-Arc, *Chlorella*-Des, and *Chlorella*-Nat cultured under different sodium nitrate concentrations. The points represent means ± SD of triplicate samples. Different letters above the bars indicate significant differences (*P* < 0.05) between growth temperatures.

The biochemical compositions of *Chlorella*-Arc, *Chlorella*-Des, and *Chlorella*-Nat were all measured at the end of exponential growth. The detailed time for *Chlorella*-Arc was on day 12, and the detailed time for both *Chlorella*-Des and *Chlorella*-Nat was day 10. There was obvious accumulation of chlorophyll in response to the increased nitrogen content for all three strains, but the chlorophyll contents of *Chlorella*-Arc and *Chlorella*-Des were generally constant after the nitrogen content reached 2.94 mmol/liter (Fig. 3a). This result was consistent with a previous report that degradation of chlorophyll could be induced by nitrogen deprivation in Chlorella protothecoides (33). Actually, chlorophyll breakdown is the most conspicuous symptom of leaf senescence and fruit ripening (34), and the character of chlorophyll breakdown in microalgae is similar to the visual degreasing observed during leaf senescence and fruit ripening. Compared with *Chlorella*-Nat, the *Chlorella*-Arc and *Chlorella*-Des strains showed higher chlorophyll contents in lower N concentrations between 0.37 and 8.82 mmol/liter. High chlorophyll content could be an indication of high photosynthetic capacity. The *Chlorella*-Arc and *Chlorella*-Des strains possibly evolved special regulation mechanisms for photosynthesis to producing chlorophyll under nutrition limitation in adapting to the extreme environments. The *Chlorella*-Nat strain has a normal regulation mechanism for producing chlorophyll. Thus, with increasing nutrition concentrations, the photosynthetic capacity of *Chlorella*-Nat is enhanced and thus the strain produces more chlorophyll. There were no significant changes in protein contents for any strains under the N-deprivation regimen, and the protein contents were just slightly higher for *Chlorella*-Des and *Chlorella*-Nat under the N-replete regimen (Fig. 3b). It was indicated that *Chlorella*-Arc had a significantly higher protein content at all treatments among the tested strains and had an obvious increased trend of protein content in response to the increased nitrogen content; this finding was consistent with a report that protein is the main product of photosynthesis in polar algae due to the lower temperatures (27).

The soluble sugar contents of the three strains did not show consistent trends with respect to N deprivation and N-replete conditions (Fig. 3c). The strains from the desert and the arctic glacier produced higher soluble sugar contents than the strains in the native lake region, indicating that this organism in desert and arctic regions tends to resist the extreme weather by increasing soluble sugar in cells, especially when sufficient nutrition is available. The seasonal and diurnal temperature fluctuations have a strong influence on the metabolic function and photosynthesis of microalgae in outdoor environments (35). For example, *Chlorella* strains in desert have a good capacity for sugar accumulation because of the diurnal temperature fluctuations (35). In addition, it has been demonstrated that microalgae in polar regions can resist the intense radiation by increasing the sugar content in cells (36), and this is consistent with the result here indicating that the strain from the arctic glacier had higher sugar content than the template native lake strain.

### Effect of nitrogen deprivation on neutral lipid and lipid content.

**FIG 4:**
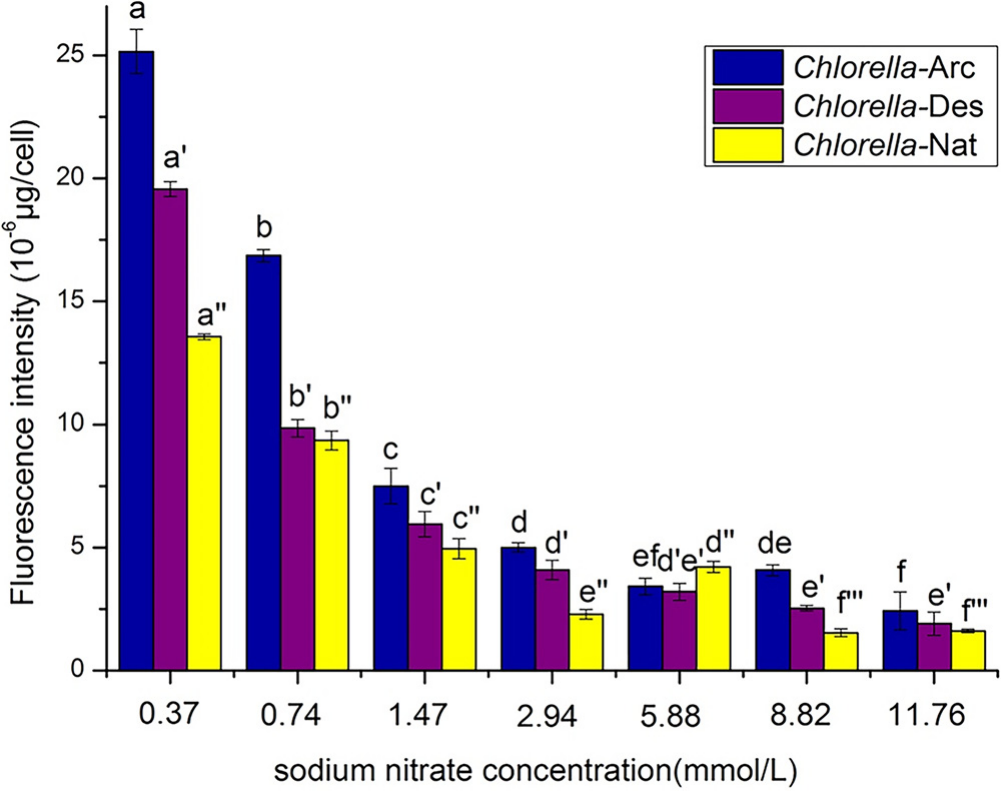
Changes in neutral lipid concentrations based on fluorescence intensity of *Chlorella*-Arc, *Chlorella*-Des, and *Chlorella*-Nat cultured under different sodium nitrate concentrations. The bar charts represent means ± SD of triplicate samples. Different letters above the bars indicate significant differences (*P* < 0.05) between growth temperatures.

The changes of neutral lipids for *Chlorella*-Arc, *Chlorella*-Des, and *Chlorella*-Nat in response to the N deprivation and N-replete conditions are shown in Fig. 4. We found that the neutral lipid accumulated obviously in response to the N deprivation for all three strains from the different environments (Fig. 4), but it was apparent that the *Chlorella*-Arc strain accumulated the highest content of neutral lipids among the three strains.

**FIG 5:**
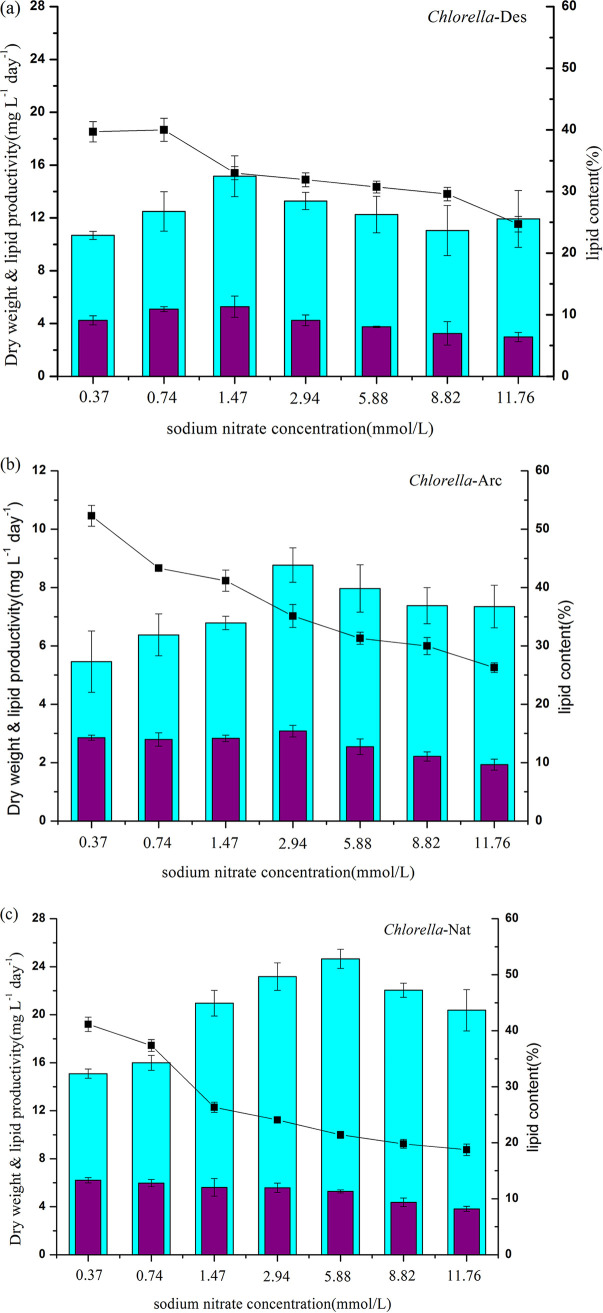
Biomass productivity, lipid productivity, and lipid content of *Chlorella*-Arc, *Chlorella*-Des, and *Chlorella*-Nat cultured under different sodium nitrate concentrations. The bar charts and points represent means ± SD of triplicate samples.

The lipid content and lipid productivity of the three *Chlorella* strains are shown in Fig. 5. It can be clearly seen that the *Chlorella* strains from the three environmental regions of polar, desert, and temperate native lake had different changes in lipid contents in response to N deprivation. For all three strains, the total lipid content under the N deprivation condition was higher than that under the N-replete condition, and this was consistent with the changes of neutral lipids. It was apparent that all three strains had higher lipid contents at the lowest nitrogen concentrations of 0.37 mmol/liter and 0.74 mmol/liter, which indicated that the N deprivation had a positive effect on the lipid accumulation. Although the *Chlorella*-Arc strain had low lipid productivity, it had the highest lipid content among the three strains under N deprivation and N-replete culture conditions, as the lipid contents reached 43% and 54% under the nitrogen concentration of 0.74 mmol/liter and 0.37 mmol/liter, respectively. The *Chlorella*-Des and *Chlorella*-Nat strains had highest lipid contents 40% and 41%, respectively. The reason for the high lipid content in *Chlorella*-Arc may be that such a strain has to achieve high utilization of nitrogen and develop special lipid accumulation processes to produce lipid for storing energy for long-term survival in extremely cold regions (37).

It was clear that among the three strains, *Chlorella*-Arc from the polar region could be a promising candidate for biodiesel feedstock, since it could accumulate higher lipid content under the N deprivation condition, and in particular the highest neutral lipid contents, which are the main materials for biofuels. Although *Chlorell*a-Arc had a low growth rate, it could be used in transgenic microalgae for biofuel production based on the genetic strain engineering strategies that offer a means to accelerate the commercialization of algal biofuels by improving the rate and total accumulation of microalga lipids (38, 39). The reason for the difference of growth rates and lipid contents of the three strains may be related to their adaptation to local environments in the long-term evolution on the genetic level. The different environmental factors could lead to their changes in physiology, which are controlled by gene regulation (40). At present, more studies are focusing on the molecular regulation of nitrogen starvation-induced lipid accumulation (11, 13, 24). For example, Goncalves and colleagues in 2016 (24) provided evidence to support the hypothesis that transcription factor ROC40 has a role in N-induced lipid accumulation, and they uncovered multiple previously unknown proteins modulated by short-term N in green algae.

### Effect of nitrogen deprivation on fatty acid composition.

Fatty acid composition, which affects the quality of biodiesel production, is strongly strain specific (41). In our research, the fatty acid compositions of the three *Chlorella* strains from arctic glacier, desert, and temperate native lake regions in response to N deprivation were well compared. Different patterns of fatty acid composition were found among the three strains from the different environmental regions (Tables 1, 2, and 3). For each strain, the fatty acid composition was also different under N deprivation and N-replete conditions. The fatty acid compositions of the three *Chlorella* strains were mainly composed of C_16_ and C_18_ fatty acids; this was similar to findings reported elsewhere (35, 42, 43). C_18:3_ (linolenic acid) contents in *Chlorella*-Arc and *Chlorella*-Nat strains were lower than 6% under all treatments (Table 2 and Table 3). Thus, the fatty acid compositions of all three *Chlorella* strains under N deprivation met the requirements of European Standard EN14214 for biodiesel production (44).

**TABLE 1:** Fatty acid compositions of *Chlorella*-Des cultured under different sodium nitrate concentrations

Fatty acid	% of total fatty acids (mean ± SD) when cultured in NaNO_3_ concn of:						
	0.37 mmol/liter	0.74 mmol/liter	1.47 mmol/liter	2.94 mmol/liter	5.88 mmol/liter	8.82 mmol/liter	11.76 mmol/liter
SFAs							
C_16:0_	21.89 ± 2.40	16.89 ± 2.12	9.64 ± 5.81	1.30 ± 0.21	11.48 ± 7.38	2.27 ± 0.09	14.16 ± 0.88
C_17:0_	9.70 ± 2.02	12.95 ± 1.39	15.70 ± 0.25	14.92 ± 1.77	16.06 ± 3.78	15.66 ± 1.27	12.72 ± 0.20
C_18:0_	1.33 ± 0.09	0.20 ± 0.34	0.36 ± 0.63	0.31 ± 0.54	0.64 ± 0.71	ND	0.34 ± 0.48
C_20:0_	ND^*a*^	0.13 ± 0.22	0.17 ± 0.30	0.44 ± 0.38	0.28 ± 0.25	ND	0.28 ± 0.39
Total	32.92 ± 4.53	30.15 ± 2.65	29.30 ± 1.38	16.97 ± 2.29	28.46 ± 2.92	17.93 ± 1.37	27.49 ± 2.31
MUFAs							
C_16:1_	3.03 ± 1.45	2.26 ± 3.28	5.55 ± 0.91	7.58 ± 0.84	3.86 ± 3.49	4.52 ± 0.08	5.52 ± 1.78
C_17:1_	2.65 ± 0.31	2.30 ± 2.01	3.26 ± 0.30	3.52 ± 0.21	2.03 ± 1.76	3.44 ± 0.02	3.45 ± 0.05
C_20:1_	27.65 ± 1.54	36.14 ± 1.70	39.71 ± 7.16	40.50 ± 0.52	37.60 ± 1.32	40.30 ± 0.11	34.76 ± 2.02
C_18:1n9t_	2.57 ± 0.53	ND	ND	ND	ND	ND	ND
C_18:1n9c_	15.66 ± 0.15	17.66 ± 2.18	17.67 ± 4.20	17.92 ± 1.07	15.92 ± 1.47	18.55 ± 0.43	16.07 ± 0.90
Total	51.57 ± 2.92	58.36 ± 2.46	59.25 ± 1.31	69.52 ± 1.73	59.40 ± 3.47	66.81 ± 0.47	59.80 ± 1.13
PUFAs							
C_18:2n6t_	2.30 ± 0.41	2.93 ± 2.62	5.20 ± 1.96	5.20 ± 0.61	4.40 ± 0.17	5.81 ± 1.32	5.29 ± 0.15
C_18:2n6c_	11.52 ± 1.18	8.56 ± 1.00	7.96 ± 1.01	8.31 ± 0.26	7.73 ± 0.61	9.45 ± 2.22	7.41 ± 0.66
Total	13.82 ± 0.78	11.49 ± 1.62	11.44 ± 0.06	13.51 ± 0.63	12.13 ± 0.77	15.26 ± 0.89	12.70 ± 0.81
UFA/SFA ratio	2.01	2.12	2.42	4.89	2.51	4.59	2.64

a ND, not detected.

**TABLE 2:** Fatty acid compositions of *Chlorella*-Arc cultured under different sodium nitrate concentrations

Fatty acid	% of total fatty acids (mean ± SD) when cultured in NaNO_3_ concn of:						
	0.37 mmol·L^−1^	0.74 mmol·L^−1^	1.47 mmol·L^−1^	2.94 mmol·L^−1^	5.88 mmol·L^−1^	8.82 mmol·L^−1^	11.76 mmol·L^−1^
SFAs							
C_16:0_	8.12 ± 0.69	3.36 ± 1.11	5.39 ± 1.42	8.86 ± 0.49	3.95 ± 0.88	25.95 ± 1.57	24.41 ± 2.68
C_20:0_	12.51 ± 1.02	17.81 ± 0.58	11.53 ± 0.66	14.54 ± 0.53	16.39 ± 0.56	2.21 ± 1.30	12.08 ± 1.27
Total	20.63 ± 2.32	21.17 ± 0.46	16.92 ± 0.28	23.40 ± 0.46	20.34 ± 0.56	28.16 ± 6.85	36.49 ± 5.69
MUFAs							
C_16:1_	12.51 ± 0.34	6.64 ± 0.46	5.27 ± 0.03	3.3 ± 0.29	5.98 ± 0.67	7.04 ± 0.79	9.81 ± 3.27
C_18:1n9t_	6.23 ± 0.28	22.47 ± 0.23	16.35 ± 1.35	15.11 ± 1.23	15.62 ± 0.86	3.50 ± 4.5	0.46 ± 0.23
C_18:1n9c_	20.46 ± 0.08	18.34 ± 0.65	20.63 ± 2.56	23.89 ± 3.45	18.47 ± 1.32	11.57 ± 6.75	13.67 ± 4.20
C_20:1_	16.33 ± 0.45	15.93 ± 0.22	16.43 ± 0.73	21.41 ± 0.70	12.91 ± 2.13	39.92 ± 0.06	33.05 ± 3.83
Total	55.53 ± 0.60	63.38 ± 3.46	58.68 ± 0.70	53.71 ± 4.56	52.89 ± 0.45	62.03 ± 11.25	56.99 ± 12.41
PUFAs							
C_18:2n6t_	17.13 ± 0.8	8.65 ± 015	7.75 ± 0.56	2.67 ± 00.56	7.52 ± 0.05	9.82 ± 8.45	7.52 ± 5.79
C_18:2n6c_	2.98 ± 0.23	3.25 ± 2.25	12.77 ± 1.38	15.67 ± 1.34	13.53 ± 0.57	2.21 ± 3.13	11.07 ± 9.33
C_18:3n3_	3.73 ± 0.19	3.55 ± 0.41	3.88 ± 0.10	4.55 ± 0.55	5.72 ± 0.61	nd^*a*^	nd
Total	23.84 ± 0.22	15.45 ± 0.87	24.40 ± 3.56	22.89 ± 2.45	26.77 ± 4.23	12.02 ± 3.20	18.60 ± 8.27
UFA/SFA ratio	3.84	5.47	3.10	3.37	2.74	2.63	2.07

a ND, not detected.

**TABLE 3:** Fatty acid compositions of *Chlorella*-Nat cultured under different sodium nitrate concentrations

Fatty acid	% of total fatty acids (mean ± SD) when cultured in NaNO_3_ concn of:						
	0.37 mmol/liter	0.74 mmol/liter	1.47 mmol/liter	2.94 mmol/liter	5.88 mmol/liter	8.82 mmol/liter	11.76 mmol/liter
SFAs							
C_16:0_	20.65 ± 1.46	28.81 ± 0.51	20.74 ± 2.52	31.4 ± 0.43	29.34 ± 0.77	16.08 ± 0.07	17.91 ± 2.14
C_18:0_	4.51 ± 0.55	2.09 ± 0.75	3.92 ± 0.79	0.63 ± 0.05	1.91 ± 0.21	10.18 ± 0.67	14.27 ± 3.21
Total	25.16 ± 0.38	30.90 ± 0.42	24.66 ± 1.67	32.03 ± 0.09	31.25 ± 0.18	26.26 ± 0.75	25.06 ± 6.81
MUFAs							
C_16:1_	1.29 ± 0.16	0.85 ± 0.07	1.27 ± 0.07	2.50 ± 0.32	0.59 ± 0.15	6.28 ± 0.53	6.66 ± 0.87
C_18:1n9t_	10.08 ± 0.90	26.70 ± 1.05	30.31 ± 0.59	18.37 ± 1.06	22.72 ± 1.28	0.45 ± 0.07	0.67 ± 0.42
C_18:1n9c_	12.26 ± 0.52	2.66 ± 1.14	5.48 ± 1.08	1.07 ± 0.19	1.16 ± 0.10	19.15 ± 0.38	17.31 ± 1.56
C_20:1_	3.99 ± 0.90	1.63 ± 0.25	5.52 ± 1.48	1.76 ± 0.48	2.09 ± 0.90	28.14 ± 0.75	19.64 ± 0.74
C_20:1n9_	18.19 ± 1.01	12.12 ± 1.55	9.64 ± 1.03	17.52 ± 0.57	19.26 ± 1.01	3.80 ± 0.54	3.91 ± 0.63
Total	45.84 ± 1.05	43.96 ± 1.36	52.22 ± 0.70	41.22 ± 3.28	45.82 ± 1.56	57.82 ± 0.07	55.29 ± 7.56
PUFAs							
C_18:2n6t_	5.95 ± 1.69	1.81 ± 0.88	3.73 ± 1.08	0.77 ± 0.19	2.70 ± 0.96	15.92 ± 0.73	ND
C_18:2n6c_	18.61 ± 1.38	21.78 ± 1.88	15.93 ± 1.60	25.38 ± 1.08	18.93 ± 1.66	ND^*a*^	ND
C_18:3n3_	4.24 ± 0.99	1.55 ± 0.39	3.46 ± 0.38	0.60 ± 0.09	1.31 ± 0.07	ND	ND
Total	28.81 ± 0.65	25.58 ± 1.45	23.12 ± 1.45	26.75 ± 2.35	22.93 ± 0.78	15.92 ± 0.74	19.65 ± 0.74
UFA/SFA ratio	2.97	2.25	3.06	2.12	2.2	2.81	2.99

a ND, not detected.

The lipids containing high amounts of saturated fatty acids (SFAs) and monounsaturated fatty acids (MUFAs) with low polyunsaturated fatty acid (PUFA) content, especially C_18:3_, are more suitable for biodiesel production, since PUFA can lead to low oxidative stability during storage (45). In our study, the sum of SFA and MUFA content was generally high (67.08 to 83.08%) in strains from all three environmental regions, and the sum of PUFA content in *Chlorella*-Arc and *Chlorella*-Des strain was generally lower than that in *Chlorella*-Nat under different sodium nitrate concentrations (Tables 1 to 3). The proportion of C_18:3_ was low (<6%) in the *Chlorella* strain from the arctic and native lake regions and was absent in the strain of the desert region (Tables 1 to 3). In addition, high UFA and SFA are important for stress resistance, since unsaturated fatty acids are more easily oxidized by oxygen radicals than are saturated fatty acids (19). *Chlorella*-Arc possessed the highest UFA/SFA ratios under the treatment of N deprivation, which indicated that this *Chlorella* strain might be the most suitable strain that not only could accumulate high lipids but also could have suitable fatty acid composition for biodiesel production under the N deprivation condition.

### Quality of RNA sequencing data and gene functional annotation.

A total of 40.37 Gb bases were generated for all strains. The clean reads quality metrics after filtering sequences that contained low-quality, adaptor-polluted, and or a high content of unknown base (N) reads, are shown in Table S1 in the supplemental material. The abundance of raw reads of the 6 strains were from 50.63 to 52.26 Mb. The clean reads ratios of all strains ranged from 84.38% to 89.74%. The number of transcripts for the six samples were 21,333, 25,771, 44,358, 51,269, 26,359, and 26,770, with a mean length ranging from 915 bp to 1,159 bp and N50 length from 1,379 bp to 1,733 bp (Table 4; see also Fig. S1 to S6). The numbers of unigenes for the six samples were 18,441, 22,145, 39,597, 45,602, 22,354, and 22,789, respectively, with a mean length of 952 bp to 1,257 bp and an N50 length of 1,457 to 1,912 (Table 4; see also Fig. S1 to S6). These reads and assembly qualities indicated that the transcriptome sequencing performed well for functional analysis.

**TABLE 4:** Quality statistics of transcripts and unigenes for each strain from three environmental regions cultured under N deprivation (1/8ND) compared to the control group (ND)

Sample	Total no.	Total length	Mean length	N50	N70	N90	% GC
Transcripts							
Nat-1/8ND	21,333	24,730,273	1,159	1,721	1,157	529	66.99
Nat-ND	25,771	28,764,323	1,116	1,721	1,131	480	66.96
Arc-1/8ND	44,358	40,980,088	923	1,433	873	378	61
Arc-ND	51,269	46,925,918	915	1,379	858	381	59.56
Des-1/8ND	26,359	28,928,276	1,097	1,730	1,140	446	66.29
Des-ND	26,770	29,424,456	1,099	1,733	1,133	447	66.15
All transcripts	195,860						
Unigenes							
Nat-1/8ND	18,441	23,193,984	1,257	1,857	1,269	605	67.04
Nat-ND	22,145	26,846,722	1,212	1,872	1,251	546	67.01
Arc-1/8ND	39,597	38,202,965	964	1,524	935	389	61.07
Arc-ND	45,602	43,438,848	952	1,457	913	392	59.65
Des-1/8ND	22,354	26,864,972	1,201	1,912	1,288	503	66.26
Des-ND	22,789	27,187,125	1,192	1,879	1,264	505	66.1
All unigenes	77,917	87,573,379	1,123	1,841	1,170	446	62.47

**FIG 6:**
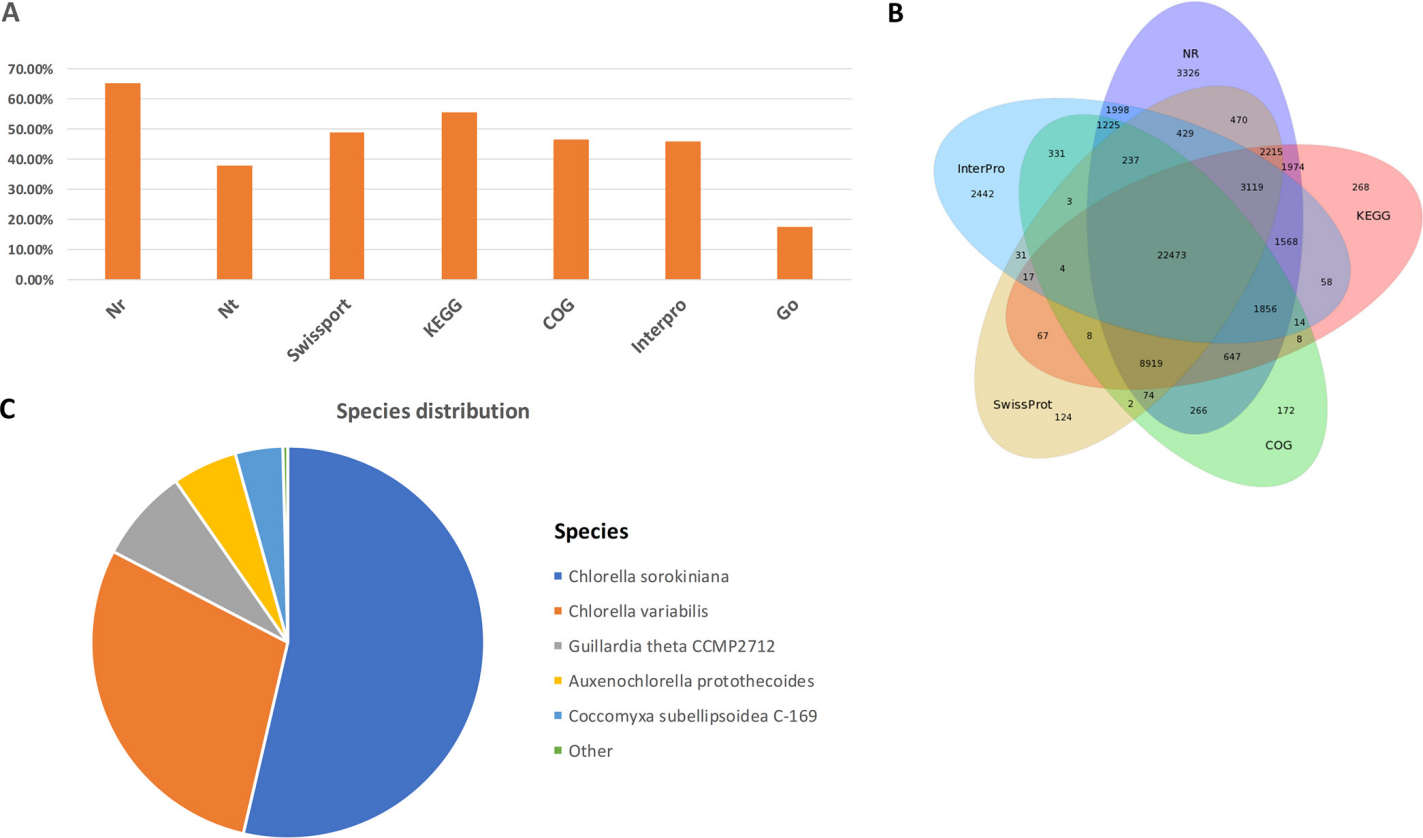
(A) Annotation showing proportions of unigenes in seven databases. (B) Annotation showing differences in seven databases for *C. sorokiniana*. (C) Species distribution for annotation of *C. sorokiniana*.

**FIG 7:**
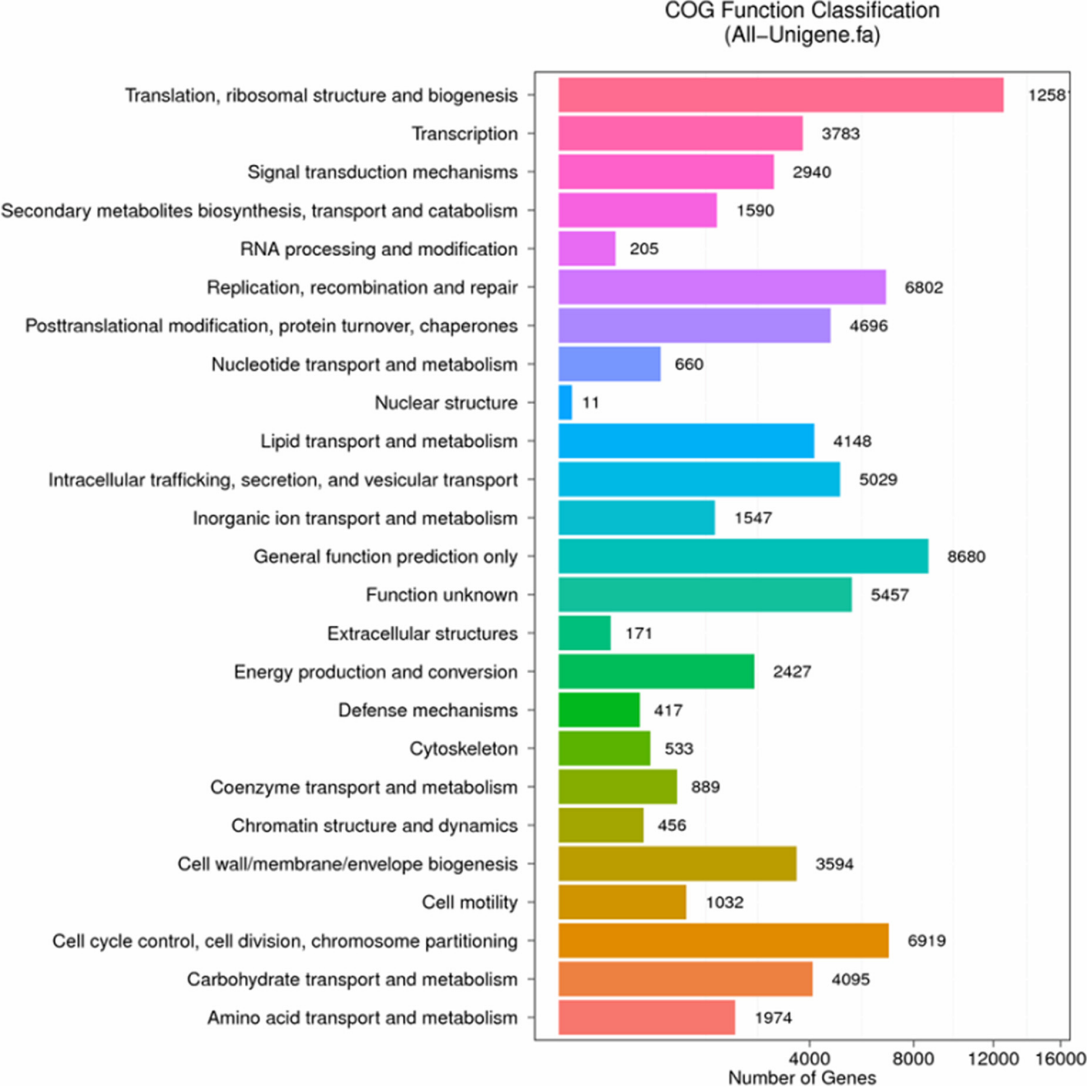
Distribution of COG function classifications for *C. sorokiniana*.

All the unigenes assembled were annotated to the NR, NT, GO, COG, KEGG, Swissprot, and Interpro databases, respectively. For all annotated unigenes, the NR, Swissprot, COG, and KEGG databases showed higher annotation proportions (Fig. 6A). Among the 7 databases, the NR database had the most unique annotation (Fig. 6B). There were 22,473 shared annotated genes among the 7 databases. The top annotated species included *C. sorokiniana*, Chlorella variabilis, Guillardia theta, Auxenochlorella protothecoides, and Coccomyxa subellipsoidea, whereas *Chlorella variabilis* and *Chlorella sorokiniana* showed higher annotation proportions (Fig. 6C). This was not strange, since the genomes of *C. sorokiniana* and *C. variabilis* strains are available in public databases (46, 47). The COG functional enrichment was performed for all unigenes where the lipid and amino acid transport and metabolism were included (Fig. 7). The translation, ribosomal structure and biogenesis, cell cycle control, cell division, chromosome partitioning, and general function predictions accounted for the top classification categories (Fig. 7). The KEGG database annotated the unigenes into five categories, including cellular processes, environmental information processing, genetic information processing, metabolism, and organism systems, whereas the translation and global and the overview maps accounted for the top annotation.

### Genetic expression patterns and pathways of differentially expressed genes.

**FIG 8:**
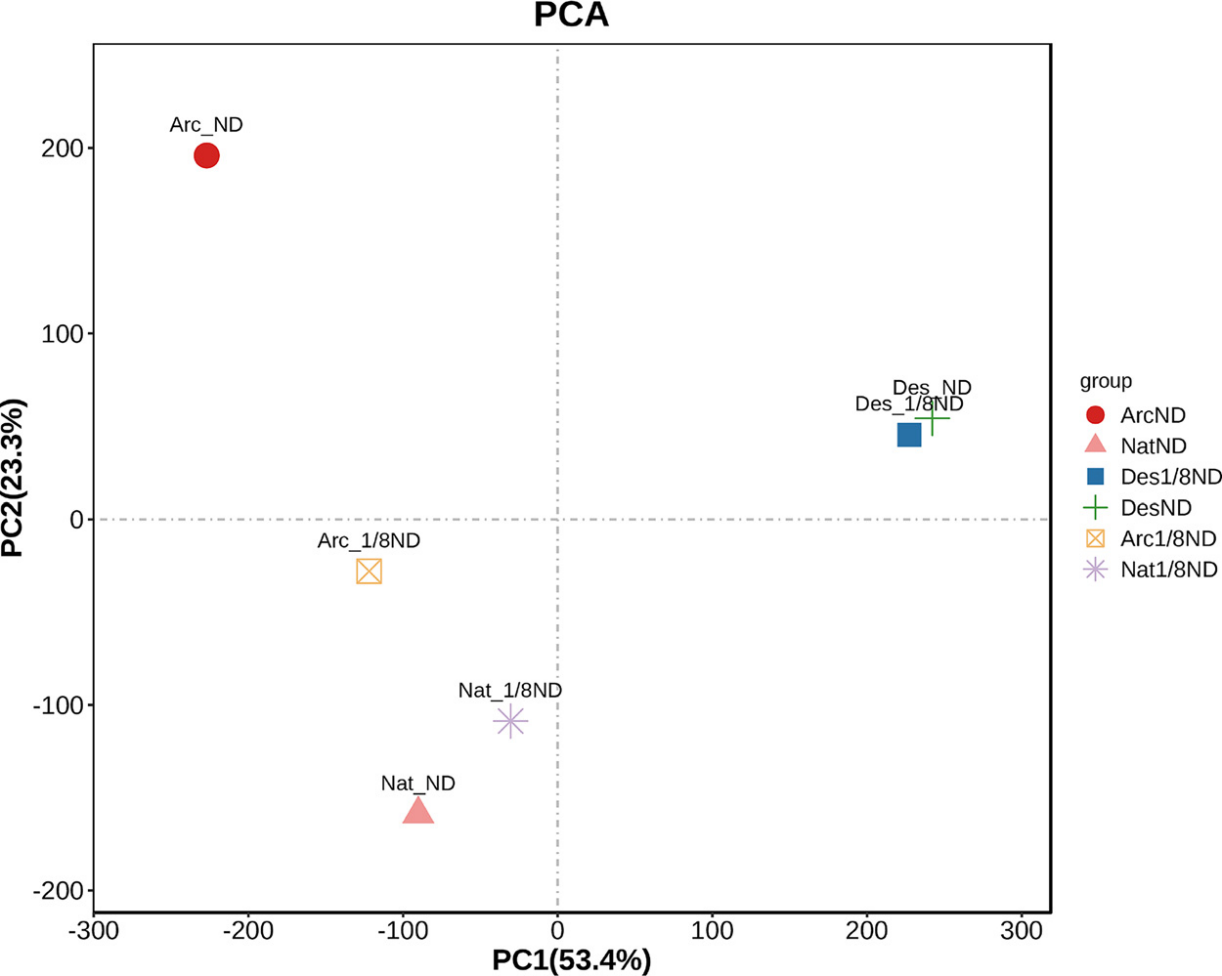
PCA plot for the differences in expression levels among the strains of the three environmental regions.

**FIG 9:**
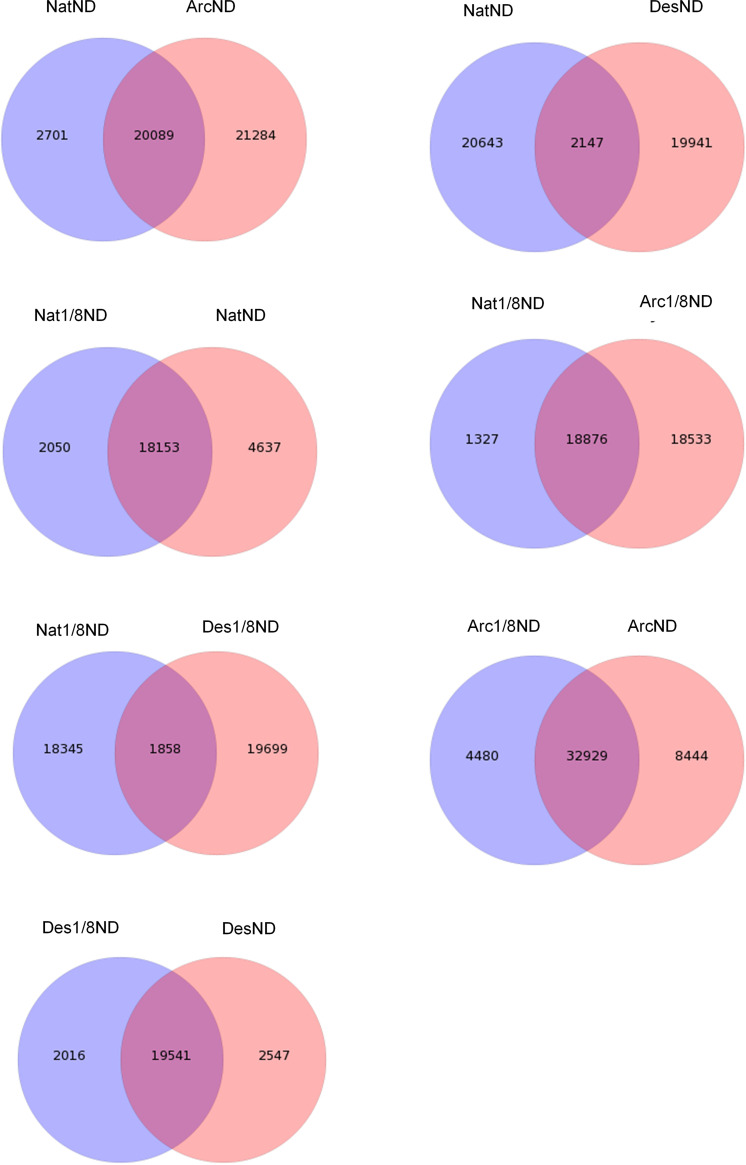
The shared and unique unigene distributions of strains among the three environmental regions.

First, the gene expression distributions of the six samples from the three strains were compared, including their shared and unique unigenes. From the principal-component analysis (PCA) plot of gene expression (Fig. 8), we noted that the strains DesND and Des1/8ND from desert clustered together closely and the NatND and Nat1/8ND strains from the native lake clustered together loosely. However, the ArcND and Arc1/8ND strains were separated. The comparison of the shared and unique unigenes among the strains are shown in Fig. 9, where the distributions of the unigenes were generally consistent with the PCA analysis. These indicated that the strains under different nitrogen stress in the desert region have similar gene expression patterns. The strains under different nitrogen stress in temperate regions also had similar gene expression patterns. However, the arctic strains under different nitrogen concentrations showed significant differentially expressed genes (DEGs).

**FIG 10:**
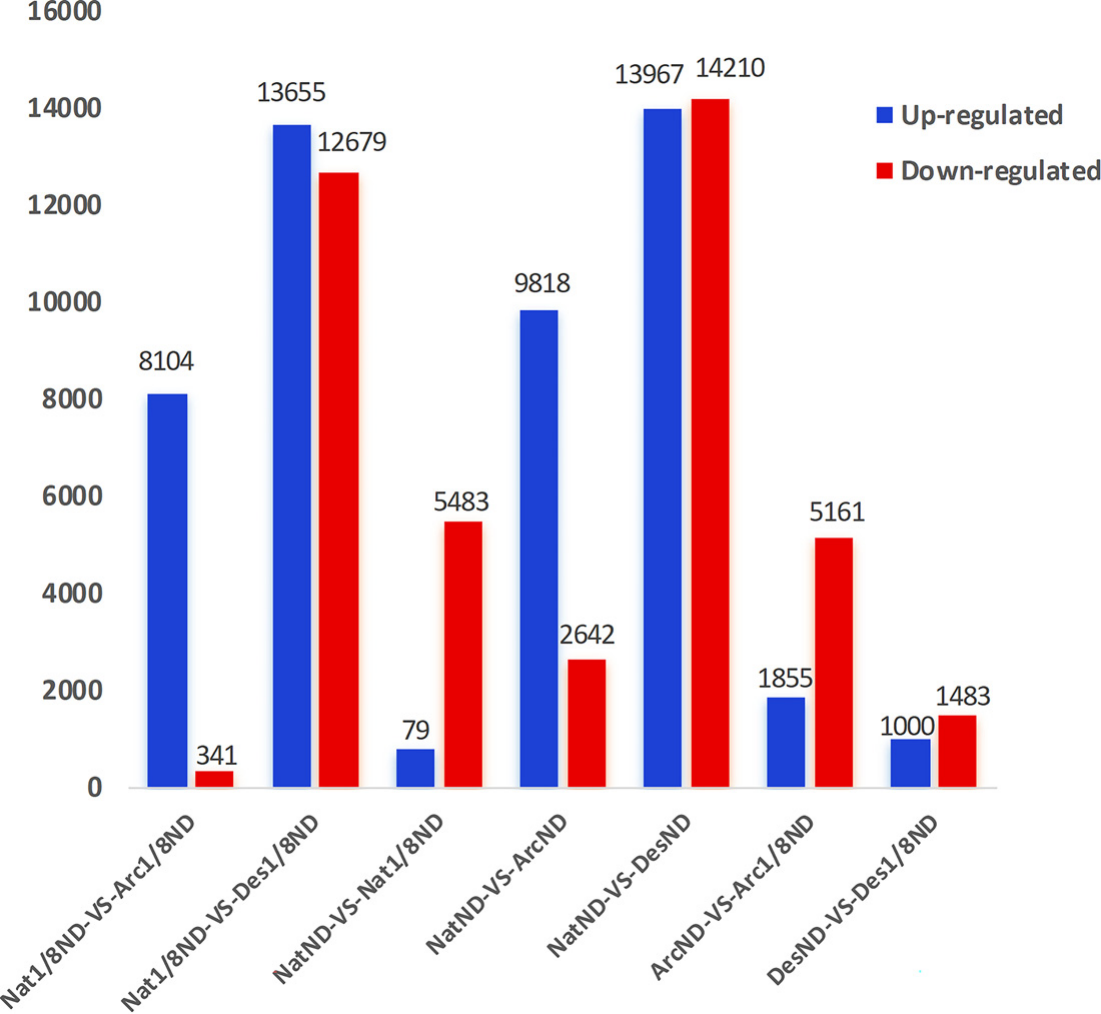
Statistics of upregulated and downregulated genes among six samples of *Chlorella-*Arc, *Chlorella-*Des, and *Chlorella-*Nat, where ND and 1/8ND indicate the concentration of NaNO_3_ (with 1 set for the control, (2.94 mmol/liter) and 1/8 times lower than the control, respectively. For example, Nat1/8ND versus Arc1/8ND comparison shows that Arc1/8ND generated more upregulated genes (blue) than downregulated genes (red) in comparison with Nat1/8ND.

**FIG 11:**
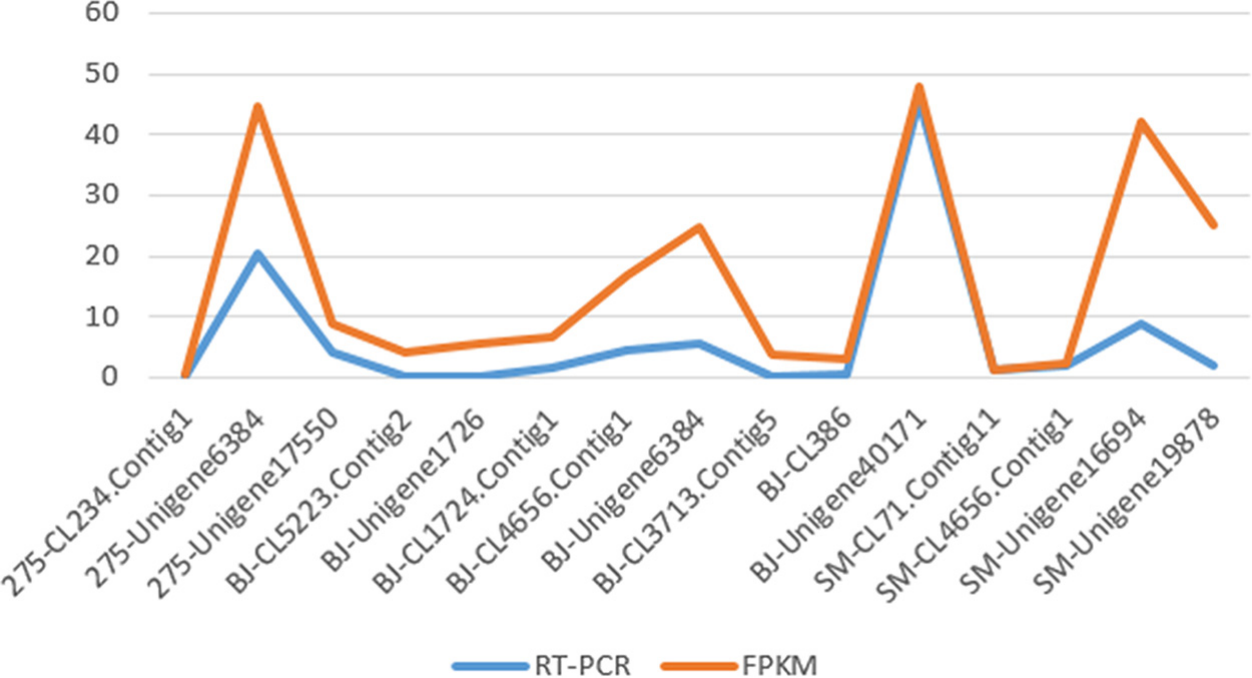
Consistency verification of relative expression levels from RT-PCR and actual expression levels from RNA-Seq.

The DEGs among the six samples of the three strains revealed upregulated and downregulated genes among them (Fig. 10). It was apparent that the Nat1/8ND versus Des1/8ND, NatND versus DesND, and NatND versus ArcND comparisons showed more DEGs, but the DesND versus Des1/8ND comparison showed fewer DEGs. The ArcND versus Arc1/8ND comparison also showed more DEGs than the DesND versus Des1/8ND comparison. This was consistent with the result from the PCA plot that showed strains from the same environmental regions had different genetic expression patterns, except for strains from the arctic region, which showed significantly different genes. These results suggest that the genetic regulated mechanisms of *C. sorokiniana* from different environmental regions differ from each other. In particular, the strains from arctic regions under the 1/8 N deprivation condition showed significantly different expression genes from the control group, which further suggested that the *C. sorokiniana* strain from the arctic region possessed a special mechanism for high lipid production (discussed below). Extreme environments like the polar region possibly make the microalgae possess special regulation mechanisms for nutrient production due to adaptive evolution (28). Multiple DEGs among the six samples were selected randomly for quantitative PCR (qPCR). The 18S gene was selected as the reference gene. In general, the statistical results indicated that the relative expression levels of the unigenes by qPCR were generally consistent with the actual expression levels (fragments per kilobase of transcript per million mapped reads [FPKM]) (Fig. 11).

**FIG 12:**
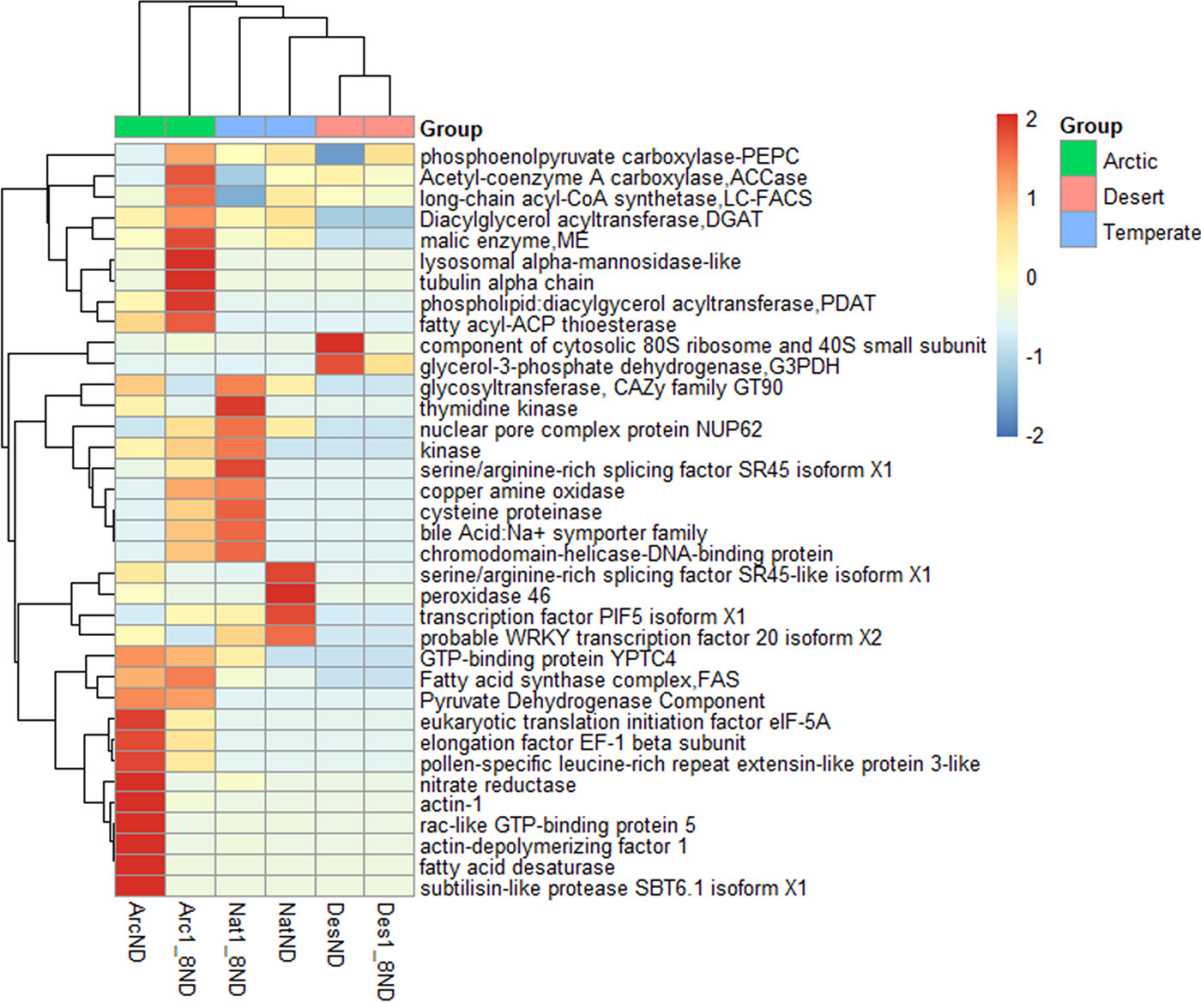
Expression comparisons of the highest DEGs among the strains of the three environmental regions.

**FIG 13:**
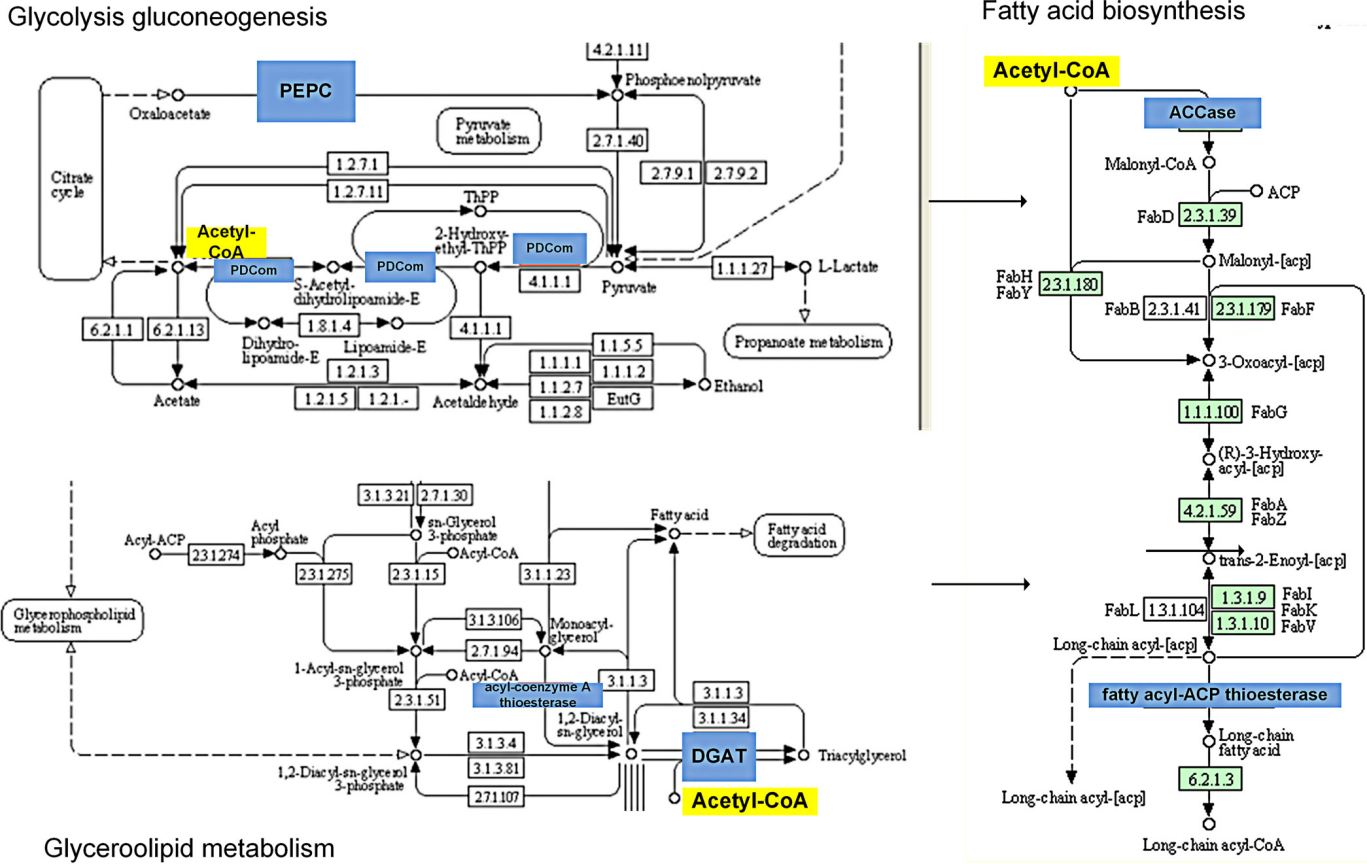
KEGG pathways of fatty acid biosynthesis, glycolysis gluconeogenesis, and glycerolopid metabolism. The genes highlighted in blue are functional catalyzing enzymes.

Among their DEGs, the most common were those obtained among the six *C. sorokiniana* samples (Fig. 12). The genes that were related to carbon and lipid metabolism were included in the DEGs, including acetyl coenzyme A (acetyl-CoA) carboxylase (ACCase), fatty acid synthase complex (FAS), diacylglycerol acyltransferase (DGAT), pyruvate dehydrogenase component (PDCom), phosphoenolpyruvate carboxylase (PEPC), long-chain acyl-CoA synthetase (LC-FACS), fatty acid desaturase (35, 40, 48, 49). These DEGs were highly expressed in the *C. sorokiniana* strain from the arctic regions (especially under 1/8 N deprivation), e.g., ACCase, LC-FACS, DGAT, PEPC, PDCom, and FAS. These findings suggested that the *C. sorokiniana* strain from the arctic region expressed more genes related to carbon and lipid metabolism to produce a high amount of lipids, consistent with our physiological results. First of all, the accumulation of large quantities of lipids in microalgae requires a continuous supply of acetyl-CoA for fatty acid biosynthesis (https://www.kegg.jp/kegg-bin/show_pathway?cvr00061) (40) (Fig. 13). In the glycolysis gluconeogenesis process of *Chlorella variabilis*, pyruvate, which is a key precursor in central carbon metabolism and lipid synthesis, can be transferred to acetyl-CoA under the catalytic action of PDCom (Fig. 13) (https://www.genome.jp/kegg-bin/show_pathway?cvr00010) (49). Meanwhile, PEPC is important for the production of pyruvate. Here, we showed that the arctic strain expressed more PDCom than the desert or temperate strains, and Arc1_8ND produced more PEPC than all other strains of the three regions (Fig. 12). The physiological results also indicated that the arctic strains produced more sugar, which can then produce more acetyl-CoA through the function of PDCom and PEPC in glycolysis gluconeogenesis. DGAT plays an important role in acetyl-CoA and triacylglycerol (TAG) biosynthesis, also referred to as catabolism of fatty acids (35, 48). In glycerolipid metabolism, TAG and acetyl-CoA are finally produced with the catalysis of DGAT (Fig. 13). In this study, DGAT was highly expressed in Arc1_8ND (Fig. 12). ACCase initiates fatty acid biosynthesis in microalgae and plays an important role for catalyzing the reaction converting acetyl-CoA to malonyl-CoA in the initial step (40). It was indicated that ACCase was highly expressed in Arc1_8ND (Fig. 12). The fatty acyl-ACP thioesterase transfers long-chain acyl-ACP to long-chain fatty acids in the last step of fatty acid biosynthesis (Fig. 13). We found that Arc1_8ND expressed the highest fatty acyl-ACP thioesterase compared to other strains.

In conclusion, the high lipid production of arctic strains is related to pathways of glycolysis gluconeogenesis, glycerolipid metabolism, and fatty acid biosynthesis. PDCom and DGAT were expressed highly in arctic strains, and thus more acetyl-CoA was produced in the glycolysis gluconeogenesis and glycerolipid metabolism. Finally, more acetyl-CoA initiated fatty acid biosynthesis through the catalytic function of ACCase and acyl-ACP thioesterase, which were highly expressed in the arctic strain.

## MATERIALS AND METHODS

### Strain isolation and identification.

The three *Chlorella* strains used in this research were isolated from glacial melted water around the Spitsbergen Bergen Islands arctic region (78°3′N, 16°6′E; *Chlorella*-Arc), desert soil from the Gurbantünggüt Desert region (45°2′N, 87°6′E; *Chlorella*-Des), and temperate native Lake Xuanwuhu, Nanjing, China (32°1′N, 118°8′E; *Chlorella*-Nat). They were first observed based on morphological characteristics by microscope after isolation. Then, molecular tools were used to identify them to the species level. Both *rbcL* and *tufA* genes were amplified and sequenced for each strain of the different regions. Phylogenetic trees were constructed by the maximum-likelihood method to identify the three strains.

### Cultivation of microalgae and experimental design.

The optimal culture temperatures for the strains from arctic glacier, desert soil, and temperate native lake were 15°C, 25°C, and 28°C, respectively, based on our previous experiments (35, 50). All the strains were grown under illumination of ~120 photons μmol/m^2^/s with a 14-h/12-h light/dark photoperiod. All *Chlorella* strains were cultivated in 250-mL flasks containing 200 mL BB medium. The initial cell density was ~5 × 10^6^ cell/mL for all three *Chlorella* strains from arctic glacier, desert soil, and temperate native lake. For the control group, the BB medium consisted of 250 mg NaNO_3_, 75 mg MgSO_4_·7H_2_O, 25 mg NaCl, 75 mg K_2_HPO_4_, 175 mg KH_2_PO_4_, 25 mg CaCl_2_·2H_2_O, 1 mL of trace elements solution which contained 11.42 mg H_3_BO_3_, 50 mg EDTA, 31 mg KOH, and 4.98 mg FeSO_4_·7H_2_O in 1 liter of fresh water, in which the concentration of NaNO_3_ was 2.94 mmol/liter. For the culture condition of N deprivation, the concentration of NaNO_3_ was set 1/8×, 1/4×, and 1/2× that for the control group (2.94 mmol/liter). For a better comparison, the concentration of NaNO_3_ was set 2×, 4×, and 8× higher than the control condition (2.94 mmol/liter) for the N-replete condition. Each condition of every strain was evaluated in triplicate. Each flask was shaken more than five times every day.

We also took steps to avoid contamination of the algae strains. Firstly, we made sure of the identification accuracy by both morphological and molecular tools. Second, during culturing, the microalgae composition was observed from time to time. We also conducted Sanger sequencing once for all the strains during culturing, to make sure that the culturing was not contaminated by other microalgae. Finally, all culturing environments were clean and isolated. All the strains from different conditions were cultured separately in their own containers.

### Measurements of growth, biochemical composition, lipid productivity, and fatty acids.

The biomass was detected based on cell number, which was counted using a hemacytometer (improved double-Neubauer). Samples were harvested by centrifugation at 8,000 × *g* for 10 min, and pellets were dried by vacuum freeze drier. Chlorophyll was analyzed spectrophotometrically after extraction with methanol overnight at 4°C in the dark (51). The soluble sugar was measured by the phenol-sulfuric acid spectrophotometric method (52). The protein was measured using the bicinchoninic acid assay (53).

The total lipid content was measured after 120-mL algal cultures at late logarithmic growth phase were harvested using a modified version of the Bligh and Dyer method (54). A rapid neutral lipid determination was performed based on the Nile red method. First, the microalgae samples were pretreated with 20% (vol/vol) dimethyl sulfoxide for 20 min at 40°C. Then, the stock solution of Nile red was prepared in acetone (0.1 mg/mL), and 15 μL Nile red dye was added into a 1-mL microalgae suspension, which was stained for 5 min. The fluorescence intensity was measured by a spectrophotometer (Molecular Devices, USA) with the excitation and emission wavelengths of 480 nm and 575 nm, respectively. The gas chromatography with mass selective detector (GC-MS; Agilent 7890A), equipped with an HP-5msi column (30 m × 0.25 mm × 0.25 μm; Agilent Technologies), was used to analyze fatty acids. Two microliters of each sample was injected into the splitless injection mode. The injection temperature and MS detector temperature were set at 250°C and 280°C, respectively. The temperature program was as follows: maintaining at the initial temperature of 50°C for 1 min, followed by increasing at a rate of 40°C/min up to 170°C, maintained for 1 min, then raising to 210°C at 18°C/min, and maintained at 210°C for 28 min. Nitrogen gas was used as the carrier gas. Finally, fatty acids were discriminated by comparison of their retention times with those of standards (Sigma). Heptadecanoic acid (C_17:0_) was used as an internal standard.

### Statistical analysis.

All samples were performed in triplicate. The mean values with their standard deviations were also determined. The significant differences were assessed by one-way analysis of variance (ANOVA) with SPSS statistical software, followed by Duncan tests. Different letters in the figures indicate significant differences between treatments (*P* < 0.05).

### RNA isolation, library construction, and RNA-Seq.

For each environmental region, arctic glacier, desert, and temperate native lake, the strains cultured in 1/8 N deprivation and the control group were selected for RNA sequencing (RNA-Seq). We did not conduct RNA-Seq of biological replicates separately for each strain. but three replicates of each strain were cultured under the same condition and were mixed first for RNA extraction and sequencing. Then, the 1/8 N deprivation condition and the control group from each environmental region were sequenced separately. Thus, a total of six samples of three strains from the three environmental regions were finally sequenced separately (see Table S1 in the supplemental material). An ethanol precipitation protocol and CTAB-PBIOZOL reagent were used for the extraction of total RNA (55). Subsequently, total RNA was qualified and quantified using a NanoDrop and Agilent 2100 bioanalyzer (Thermo Fisher Scientific, MA, USA).

Oligo(dT)-attached magnetic beads were used to purify mRNA. Purified mRNA was fragmented into small pieces with fragment buffer, which was then used as a template to generate the first-strand cDNA for a first-strand reaction system by PCR, followed by a second-strand cDNA synthesis. The reaction product was purified by magnetic beads. Next, A-Tailing mix and RNA index adapters were added, and mixtures were incubated to end-repair the cDNA fragments with adaptors amplified by PCR, and the products were purified by Ampure XP Beads. The product was validated on the Agilent Technologies 2100 bioanalyzer for quality control. The qualified library was amplified on cBot to generate the cluster on the flow cell. And the amplified flow cell was sequenced single-end on the HiSeq X-10 platform (BGI-Shenzhen, China).

### RNA-Seq data preprocessing.

For each of the six samples, their clean reads were obtained after filtering the raw reads via SOAPnuke (v1.4.0) by removing reads containing adaptors, poly(N), or low-quality reads (56). Then, the clean reads of each of the six samples were assembled separately (Table S1). Trinity (v2.0.6) (57) was used to assemble the clean reads, and Tgicl (v2.0.6) (58) was used to perform clustering and eliminate redundant data in the assembled transcripts to obtain unique genes. We compared the unigene distributions of seven sets: NatND versus ArcND, NatND versus DesND, Nat1/8ND versus NatND, Nat1/8ND versus Arc1/8ND, Nat1/8ND versus Des1/8ND, Arc1/8ND versus ArcND, and Des1/8ND versus DesND, including their shared and unique unigenes, where ND and 1/8ND indicate the concentration of NaNO_3_ in the control (2.94 mmol/liter) and 1/8 times lower than the control, respectively. Then, based on these sets, their assembled transcripts were processed for further annotation and expression analysis. Clean reads were mapped to the assembled unique genes by Bowtie2 (v2.2.5) (59), and the expression levels of genes were calculated by RSEM (v1.2.8) (60) and normalized to the FPKM. Functional annotation of genes was achieved by mapping genes to different databases (NT, NR, GO, COG, KEGG, Swiss-Prot, and InterPro) using the software BLAST (v2.2.23) (61). GO annotation was performed by Blast2GO (v 2.5.0) with NR annotations. PossionDis (62) was used to detect DEGs, and DEGs with a fold change of >2 or <−2 and a false-discovery rate (FDR) of <0.001 were considered significantly differentially expressed genes. GO enrichment analysis and KEGG enrichment analysis were performed using Phyper, a function of R. The significant levels of terms and pathways were corrected by *Q* value with a rigorous threshold (*Q* < 0.05).

### Validation of DEGs by qRT-PCR.

The confidence of the high-throughput transcriptome sequencing was further validated by quantitative real-time PCR (qRT-PCR). A total of 16 random differentially expressed genes were selected for qPCR using the SG Fastq PCR master mix (High Rox), and the primer Premier 5.0 was used for designing primers for these genes. PCRs were carried out in a total volume of 20 μL, including 10 μL of SybrGreenq PCR master mix. PCR conditions for all primer sets were 95°C for 3 min, 45 cycles of 95°C for 7 s, 57°C for 10 s, and 72°C for 15 s, and this was followed by disassociation curve analysis in ABI Steponeplus (Applied Biosystems, Foster City, CA, USA). The relative gene expression levels of the samples were calculated by the 2^−ΔΔ^*^CT^* threshold cycle method, and these levels were normalized using the 18S mRNA level. The SPSS was used to determine whether the expression data included any significant differences at the *P* < 0.05 level. The fold changes of these genes were calculated via FPKM, and the gene’s log_2_ fold change values from qRT-PCR and RNA-Seq were used for graphical presentations.

### Data availability.

The next-generation sequencing (NGS) data of all samples obtained in this study have been deposited with NCBI. The SRA accession for the NGS sequences is PRJNA797923. The submission information for each sample mainly includes the reads sequences in fastq form, the sampling location, and the sequence platform.
