# Supplementary material for: Physiological and Genetic Regulation for High Lipid Accumulation by Chlorella sorokiniana Strains from Different Environments of an Arctic Glacier, Desert, and Temperate Lake under Nitrogen Deprivation Conditions
Source: Microbiol Spectr. 2022 Oct 6;10(5):e00394-22. doi: 10.1128/spectrum.00394-22 (PMC9603131; doi:10.1128/spectrum.00394-22)
Supplement: Supplemental file 2 — Figure S1-S6. Download spectrum.00394-22-s0002.pdf, PDF file, 0.9 MB [file spectrum.00394-22-s0002.pdf]

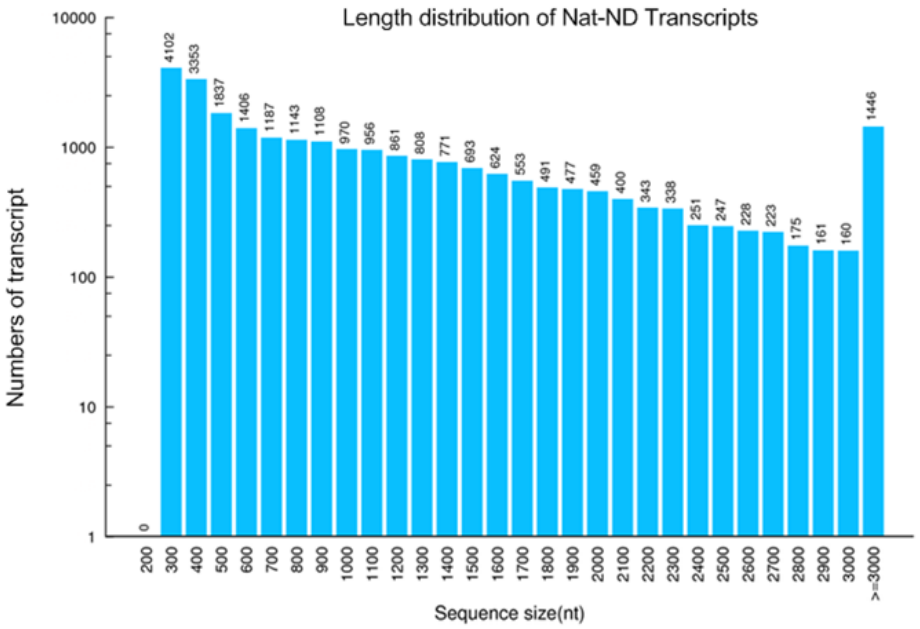

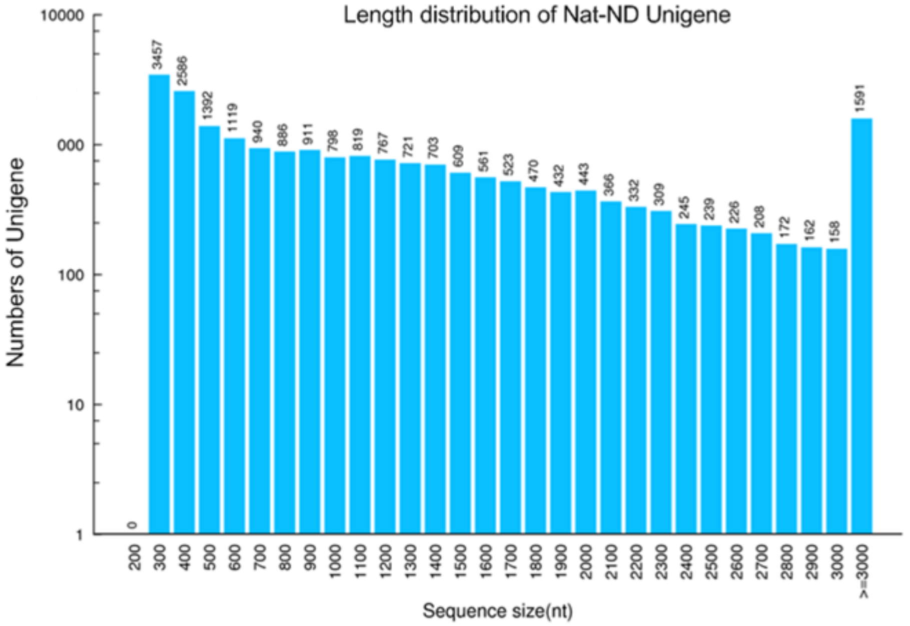

Figure S1. The number of unigenes for *Chlorella*-Nat, where Nat-ND means the concentration of NaNO3 was set in control.

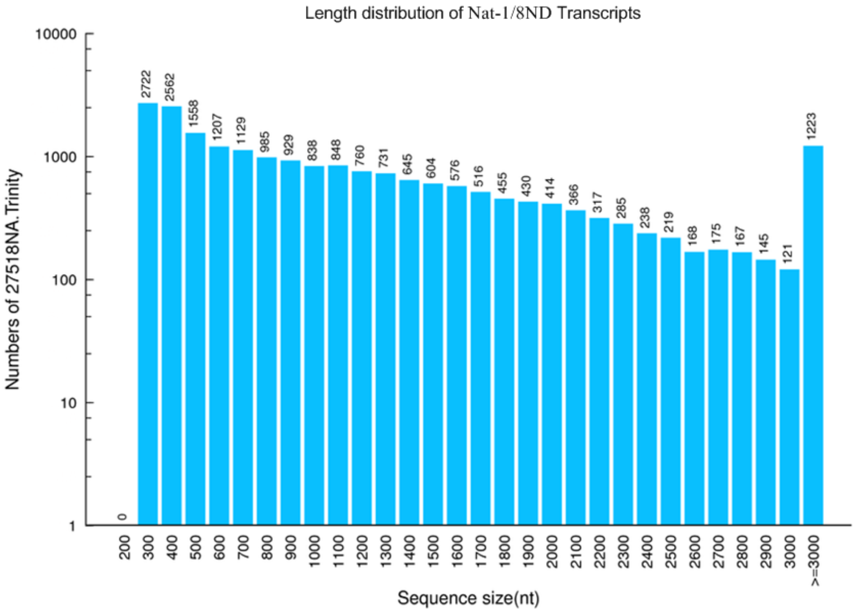

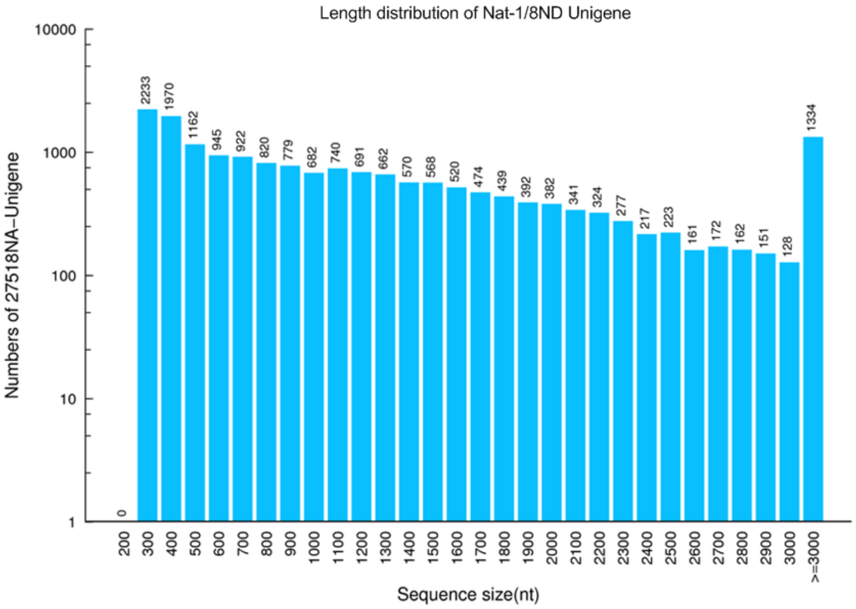

Figure S2. The number of unigenes for *Chlorella*-Nat, where Nat-1/8ND means the concentration of NaNO3 was set in 1/8 times lower than the control.

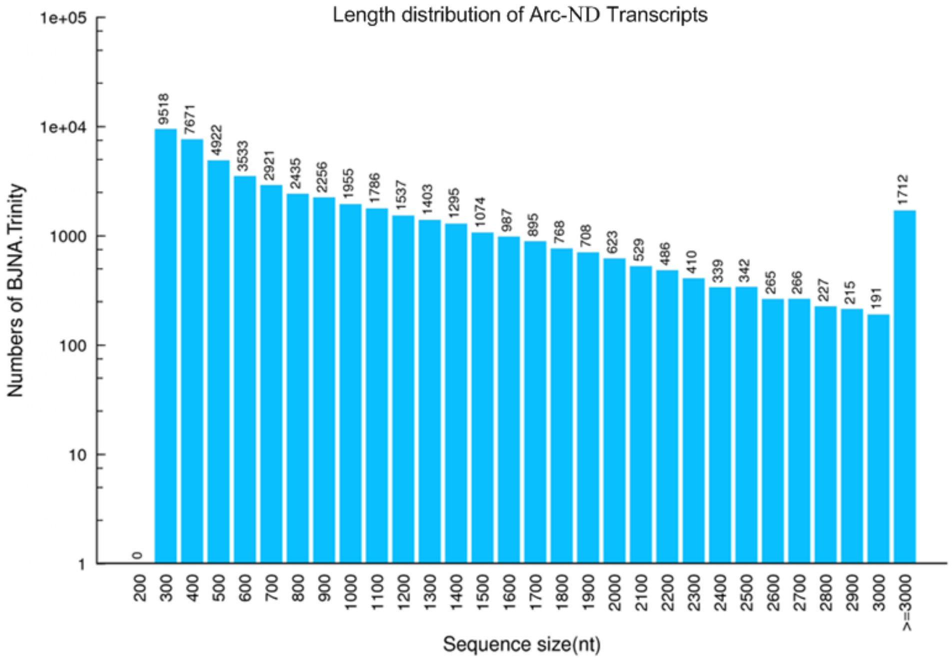

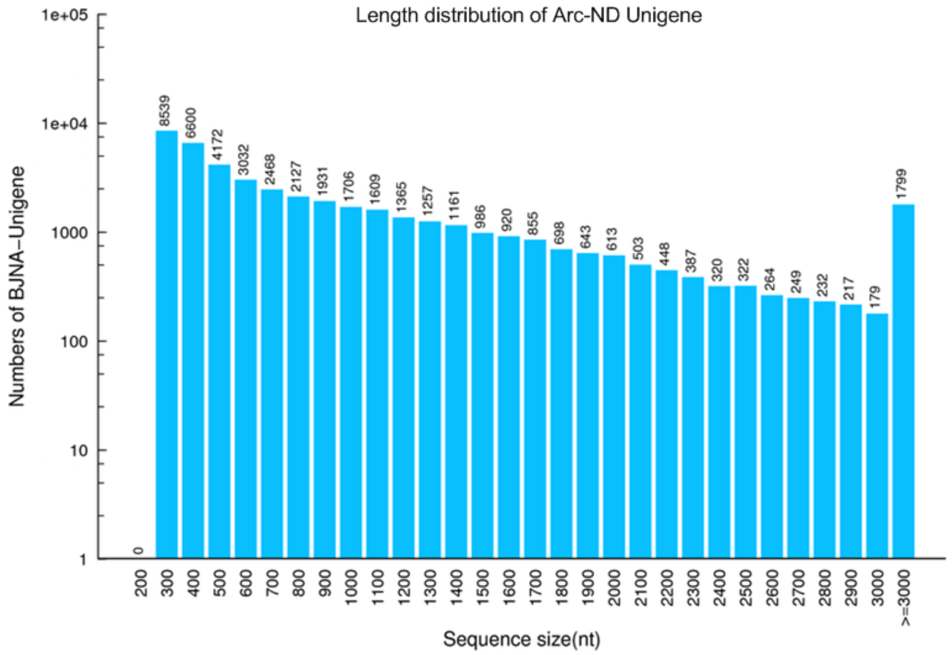

Figure S3. The number of unigenes for *Chlorella*-Arc, where Arc-ND means the concentration of NaNO3 was set in control.

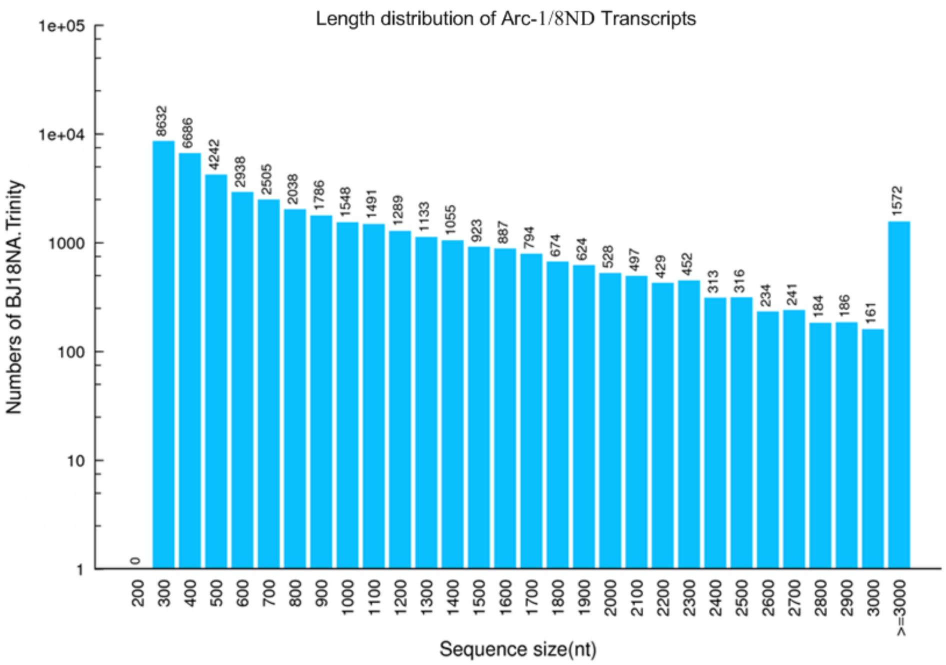

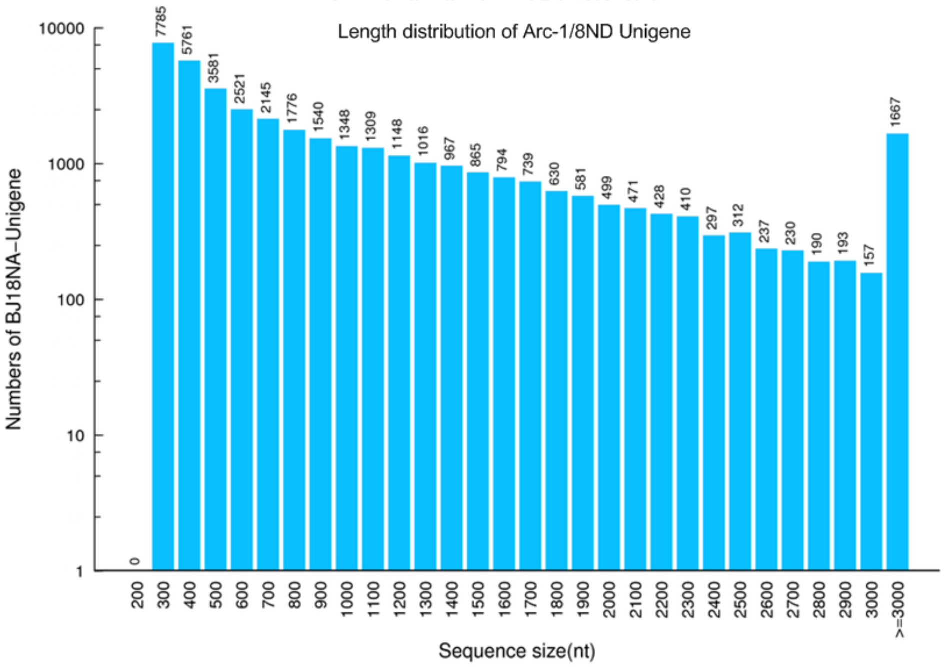

Figure S4. The number of unigenes for *Chlorella*-Arc, where Arc-1/8ND means the concentration of NaNO3 was set in 1/8 times lower than the control.

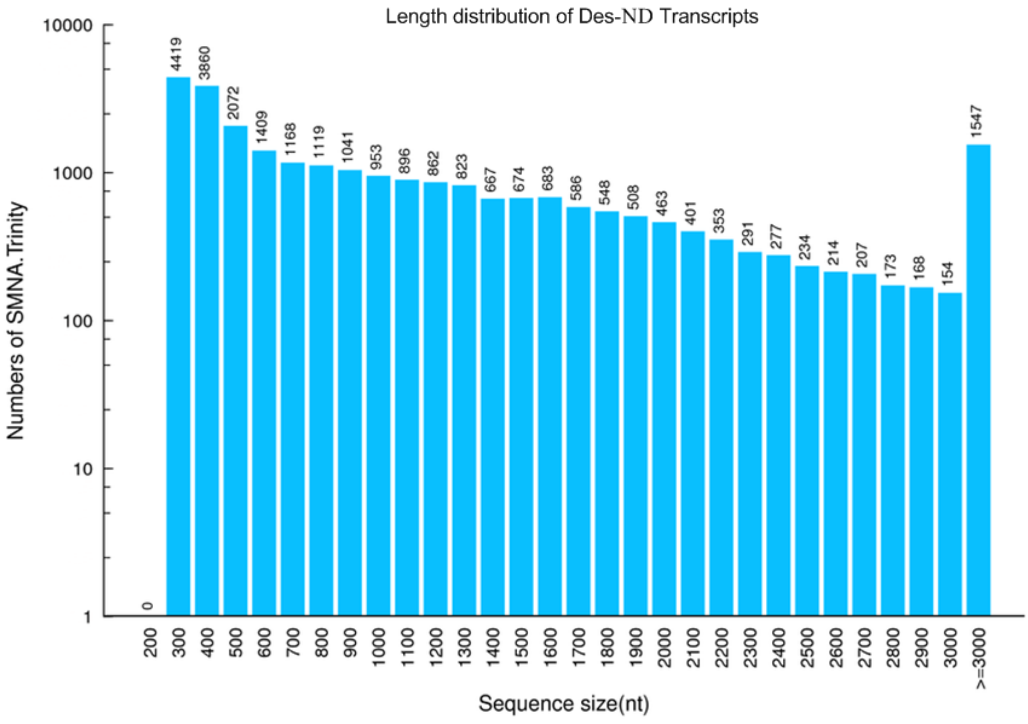

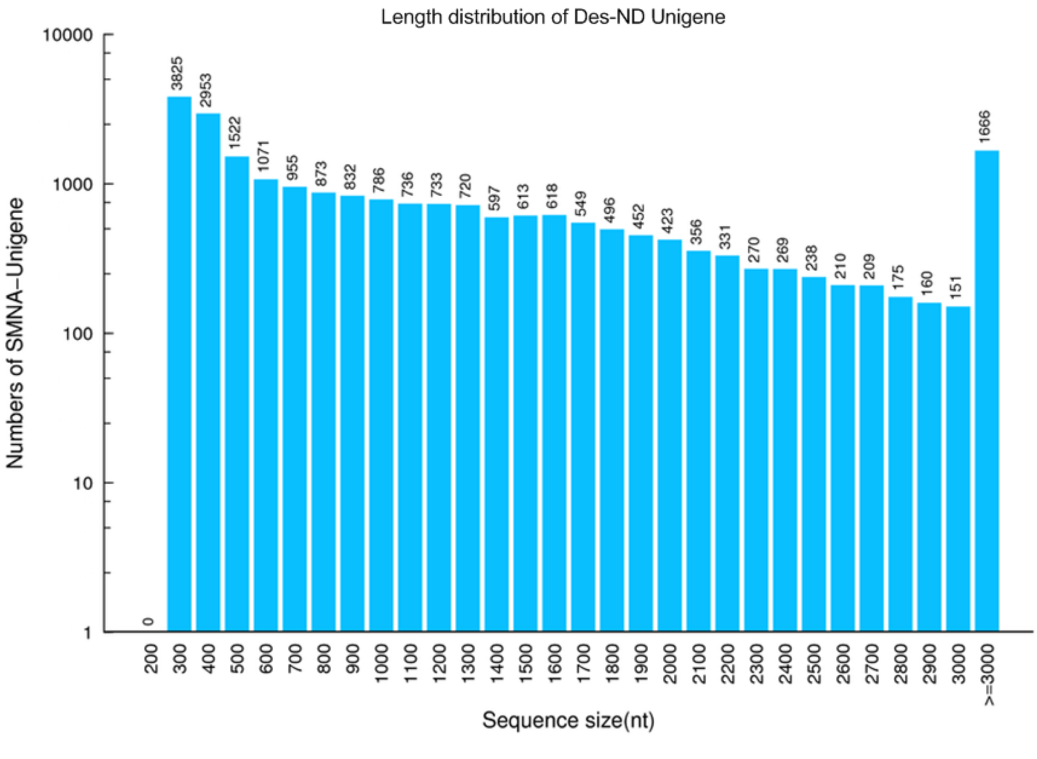

Figure S5. The number of unigenes for *Chlorella*-Des, where Des-ND means the concentration of NaNO3 was set in control.

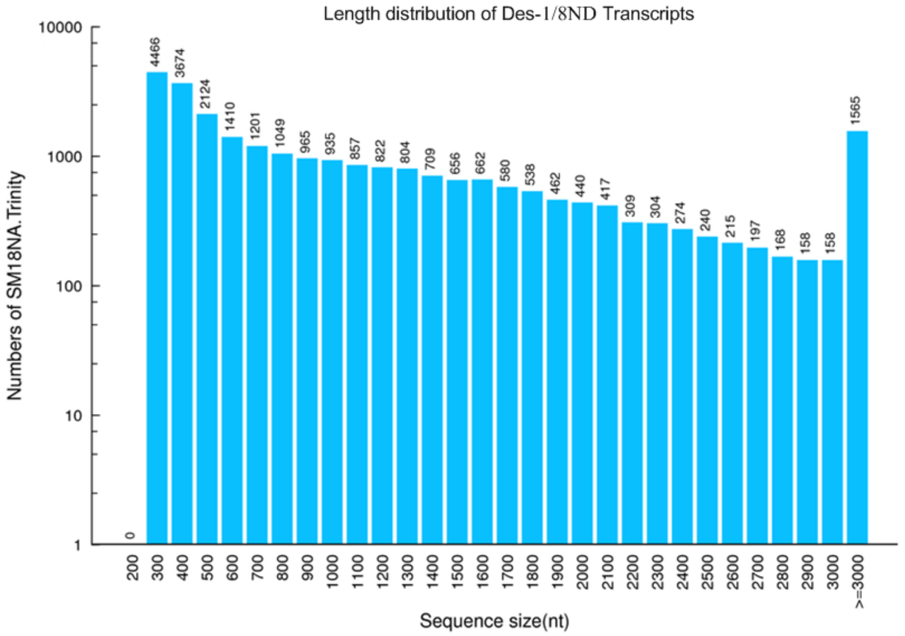

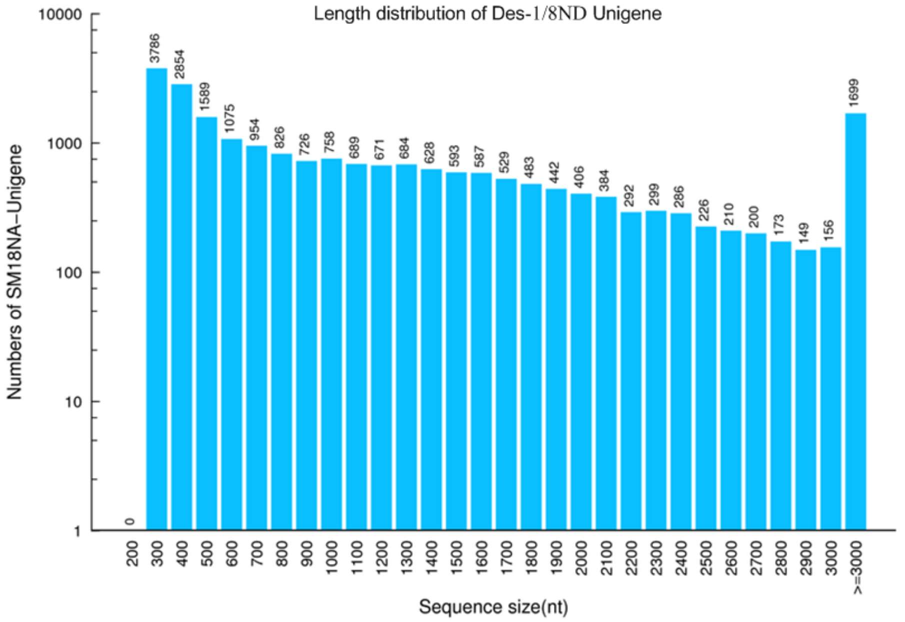

Figure S6. The number of unigenes for *Chlorella*-Des, where Des-1/8ND means the concentration of NaNO3 was set in 1/8 times lower than the control.
